# A Multimodal Haptic Feedback Interface with Thin‐Film Compliant Mechanism

**DOI:** 10.1002/advs.76412

**Published:** 2026-07-08

**Authors:** Jingjing Wan, Emanuele Nicotra, James Davies, Kefan Zhu, Quang Anh Nguyen, Sinuo Zhao, Chi Cong Nguyen, Bibhu Sharma, Adrienne Ji, Hermione Truong, Patrick Pruscino, Tan Huynh, Phuoc Thien Phan, Hoang‐Phuong Phan, Nigel Hamilton Lovell, Thanh Nho Do

**Affiliations:** ^1^ School of Biomedical Engineering Faculty of Engineering UNSW Sydney Sydney New South Wales Australia; ^2^ School of Mechanical and Manufacturing Engineering Faculty of Engineering UNSW Sydney Sydney New South Wales Australia; ^3^ Tyree Institute of Health Engineering (IHealthE) UNSW Sydney Sydney New South Wales Australia

**Keywords:** compliant mechanism, electromagnetic actuator, multimodal haptic devices

## Abstract

Multimodal cutaneous haptic interfaces can deliver multiple forms of mechanical stimulation and trigger different mechanoreceptors, enabling richer haptic information and realistic tactile sensations. Such interfaces have broad potential in healthcare, entertainment, and education. Electromagnetic (EM) actuators are particularly attractive for multimodal haptic systems due to their compact size, high controllability, and fast dynamic response. However, existing EM‐based multimodal haptic devices struggle to simultaneously achieve large displacement, high output force, and effective constraint of unwanted degrees of freedom. To address this research gap, this paper presents a new thin‐film compliant mechanism that exhibits high flexibility in translational motion along the X, Y, and Z axes while maintaining high stiffness against rotations about the *X* and *Y* axes. The new design enables the development of an electromagnetic multimodal haptic module capable of delivering indentation, stretch, and vibration stimuli, with the ability to superimpose vibration onto skin stretch. By integrating such two haptic modules into a wearable haptic array and employing a spatiotemporal encoding strategy, four‐directional haptic cues can be effectively conveyed on hairy forearm skin, achieving a mean discrimination accuracy of 79.5%. Finally, a vision‐guided human‐in‐the‐loop haptic assistance system is implemented to demonstrate feasibility in assisting visually impaired users with daily tasks.

## Graphical abstract

Multimodal haptic devices can trigger different mechanoreceptors, enabling richer haptic information. This study presents an electromagnetic multimodal haptic device capable of delivering indentation, stretch, and vibration stimuli. The device is enabled by a novel polymer thin‐film compliant mechanism that provides high translational compliance along the *X*, *Y*, and *Z* axes while maintaining high rotational stiffness about the *X* and *Y* axes.

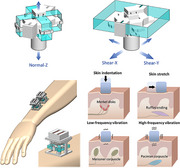

## 1 Introduction

Cutaneous haptic feedback interfaces are devices that directly act on the skin to deliver tactile information or render tactile interactions for virtual environments through mechanical, electrical, or thermal stimulation [1, 2, 3]. Such haptic technologies have been widely applied in virtual and augmented reality (VR/AR) [4, 5, 6, 7], teleoperation of robotic systems [8, 9, 10, 11], rehabilitation training [12, 13, 14, 15, 16], sensory substitution [17, 18], navigation [19, 20, 21, 22], and communication [23, 24, 25]. Mechanical stimulation is the most important modality in cutaneous haptic feedback, as it activates cutaneous mechanoreceptors by inducing skin deformation. These mechanoreceptors primarily include four types: Merkel disks, Ruffini endings, Meissner corpuscles, and Pacinian corpuscles. Merkel discs are sensitive to static normal compression, Ruffini endings respond to skin stretch, whereas Meissner and Pacinian corpuscles are particularly sensitive to low‐frequency and high‐frequency vibrations, respectively [26, 27]. To enable haptic devices to trigger as many mechanoreceptors as possible and thereby achieve higher information bandwidth and more realistic rendering of virtual environments, recent research has increasingly focused on the development of multimodal mechanical haptic interfaces [28]. Single‐modality haptic devices typically induce only one type of skin deformation, and their limited kinematic degrees of freedom are insufficient to support complex tactile encoding. In contrast, multimodal haptic devices can switch between different forms of mechanical stimulation and even deliver multiple stimuli simultaneously (shear forces superimposed with vibration). Previous studies have employed a variety of actuators to realize multimodal mechanical stimulation. The motor is one of the most stable actuators, being easy to control and integrate [29, 30, 31, 32, 33]. Its internally integrated reduction gear set enables large force output. However, it also results in slow dynamic response and limited capability for high‐frequency vibration. Fluid‐driven actuators (pneumatic or hydraulic) provide compliant and safe output, but they typically rely on bulky pump‐valve systems [16, 34, 35, 36, 37, 38, 39, 40, 41]. The shape memory alloy (SMA), on the other hand, has the advantages of compact size and high force density, but its slow response time due to thermal actuation limits its application to low‐frequency haptic feedback [42, 43]. Electrostatic actuators offer low power consumption and fast response. However, the high operating voltages required raise safety concerns related to dielectric breakdown [44].

Compared with these actuators, electromagnetic (EM) actuators exhibit a more balanced performance. They exhibit fast dynamic responses and can generate high‐frequency vibrations. Moreover, their control and power systems can be made compact, making them well‐suited for wireless wearable haptic devices. The Lorentz forces generated by EM actuation can be precisely controlled, allowing accurate modulation of the output mechanical stimuli. Multimodal haptic devices based on EM actuators typically comprise three key components: an EM coil array, a permanent magnet, and a passive mechanical structure. By regulating the magnitude and direction of the currents flowing through the EM coils, spatially varying magnetic fields can be generated. The permanent magnet experiences Lorentz forces within the magnetic field, and the passive structure constrains the magnet's motion and ensures that the forces delivered to the skin remain well controlled.

Although the EM actuator determines the magnitude and direction of the generated electromagnetic forces, the passive structure governs how these forces are effectively transmitted to the skin during tactile interaction. For multimodal haptic devices, the passive structure enhances haptic interaction controllability by mechanically decoupling the allowable motion modes. Therefore, the design of the passive mechanical structure is equally important as the actuator itself. An ideal passive structure should satisfy the following requirements: (1) restoring the permanent magnet to its initial central position after the tactile stimulation ends, (2) minimizing mechanical resistance during permanent magnet motion to enable a larger output force and displacement on the skin, (3) effectively constraining unwanted torsional rotation of the permanent magnet during shear motion. Although several passive mechanism designs have been proposed in previous studies, each still suffers from inherent limitations. For example, Ha et al. directly attached the permanent magnet to the skin using double‐sided adhesive tape and used an arm structure to suppress undesired torsion during shear motion [28]. In this system, the skin acted as a passive mechanism to constrain the movement of the permanent magnet. Although this design effectively reduced the device's size, it could cause skin abrasions and user discomfort, particularly when the device was removed from hairy skin. Chen et al. used a compliant mechanism composed of four kirigami springs to support the permanent magnet [45]. However, this design was unable to effectively constrain out‐of‐plane torsional rotations during shear actuation. Kim et al. implemented a rigid passive guide mechanism using titanium rods and silicone tubes, which restricted the motion range of the permanent magnet [46]. Furthermore, the tactors used to induce normal and shear forces in this design were not integrated into a single unit. These examples show that existing EM multimodal haptic devices lack an effective passive mechanical structure that simultaneously enables low mechanical resistance, large translational displacement, and strong constraint of torsional motion.

**FIGURE 1:**
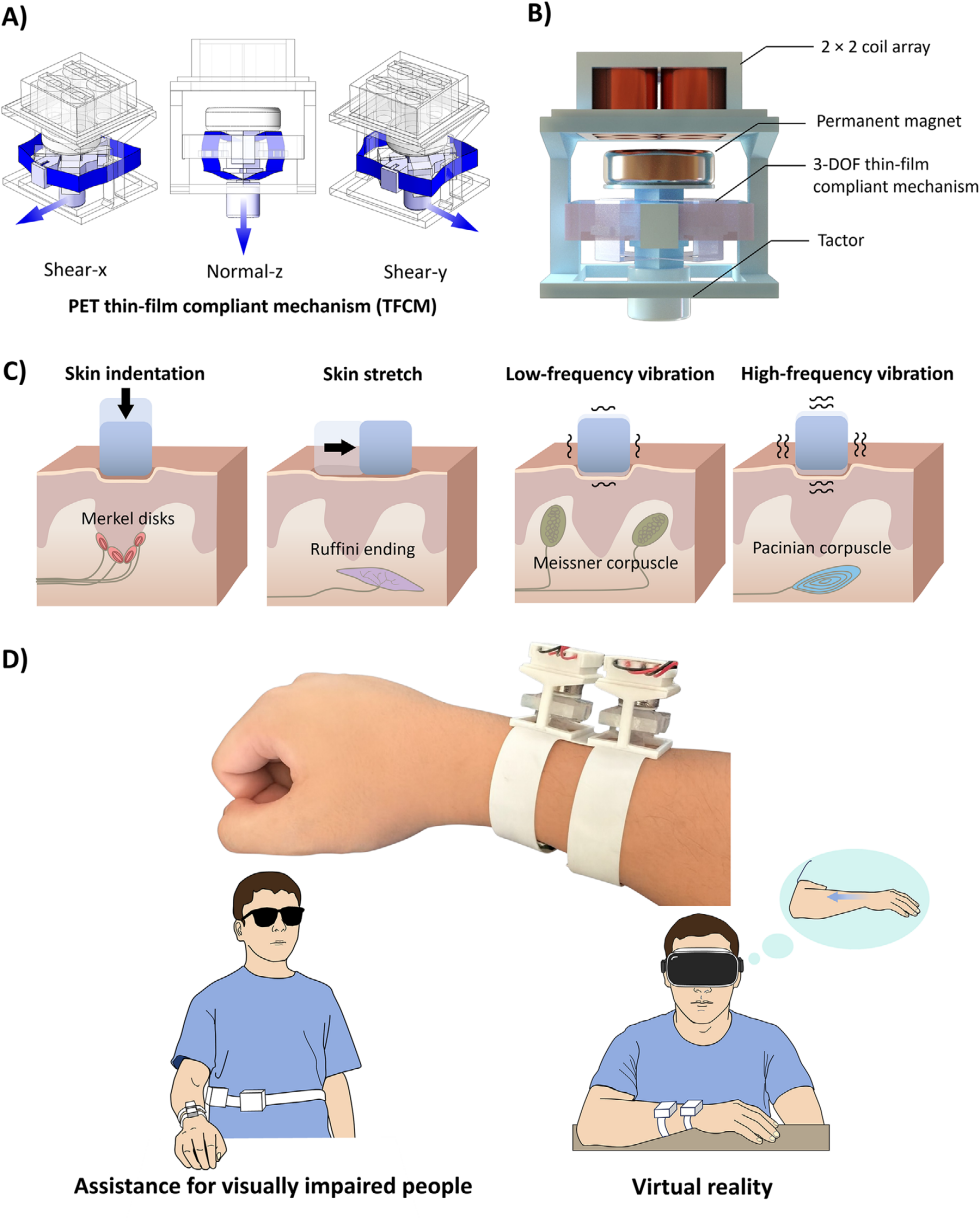
Overview of the multimodal electromagnetic haptic device. (A) The 3‐DOF thin‐film compliant mechanism (TFCM) enables translational motion along the *X*, *Y*, and *Z* axes, while effectively constraining rotation about both the X and Y axes. (B) Four core components of the haptic device: an electromagnetic coil array, a permanent magnet, a 3‐DOF compliant mechanism, and a silicone‐covered tactor. (C) Multimodal tactile stimuli can be generated on the human skin. (D) Two potential application scenarios of the 1 × 2 wearable multimodal haptic array.

To address this research gap, this work proposes a novel three‐degree‐of‐freedom (DOF) polymer thin‐film compliant mechanism (Figure 1A) that allows the movement of the permanent magnet with low resistance and large displacement along the *X*, *Y*, and *Z* axes, while effectively constraining the rotational degrees of freedom about the *X* and *Y* axes. Building on this new compliant mechanism, a wireless, wearable EM multimodal haptic module (Figure 1B) capable of delivering three types of mechanical tactile stimuli on the skin: indentation, stretch, and vibration (Figure 1C) is developed. Additionally, a wearable haptic array device on the forearm using two multimodal haptic modules is also created. By employing a spatiotemporal encoding strategy, this haptic array device can clearly convey directional tactile signals, showing potential for future applications in assisting visually impaired users and rendering virtual environments (Figure 1D).

## 2 Results

### 2.1 Design and Fabrication

As shown in Figure 1B, the haptic module consists of four components: a 2 × 2 electromagnetic coil array, an axially magnetized N52 permanent magnet, a 3‐DOF Polyethylene Terephthalate (PET) thin‐film compliant mechanism (TFCM), and a tactor. By independently controlling the magnitude and direction of electric currents in the four electromagnetic coils, the resulting Lorentz force acting on the N52 permanent magnet can be precisely modulated, thereby enabling 3D motion of the magnet. The permanent magnet is bonded to the upper end of the TFCM using a high‐strength adhesive, and the lower end of the TFCM connects to the tactor. Consequently, the force applied to the skin is the resultant of the Lorentz force and the elastic restoring force. The TFCM can be divided into a 2‐DOF compliant mechanism and a 1‐DOF compliant mechanism. The 2‐DOF compliant mechanism enables in‐plane shear forces along the X and Y axes, whereas the 1‐DOF compliant mechanism enables out‐of‐plane normal forces along the Z axis. Movie S1 shows the motion of the TFCM in all three directions.

**FIGURE 2:**
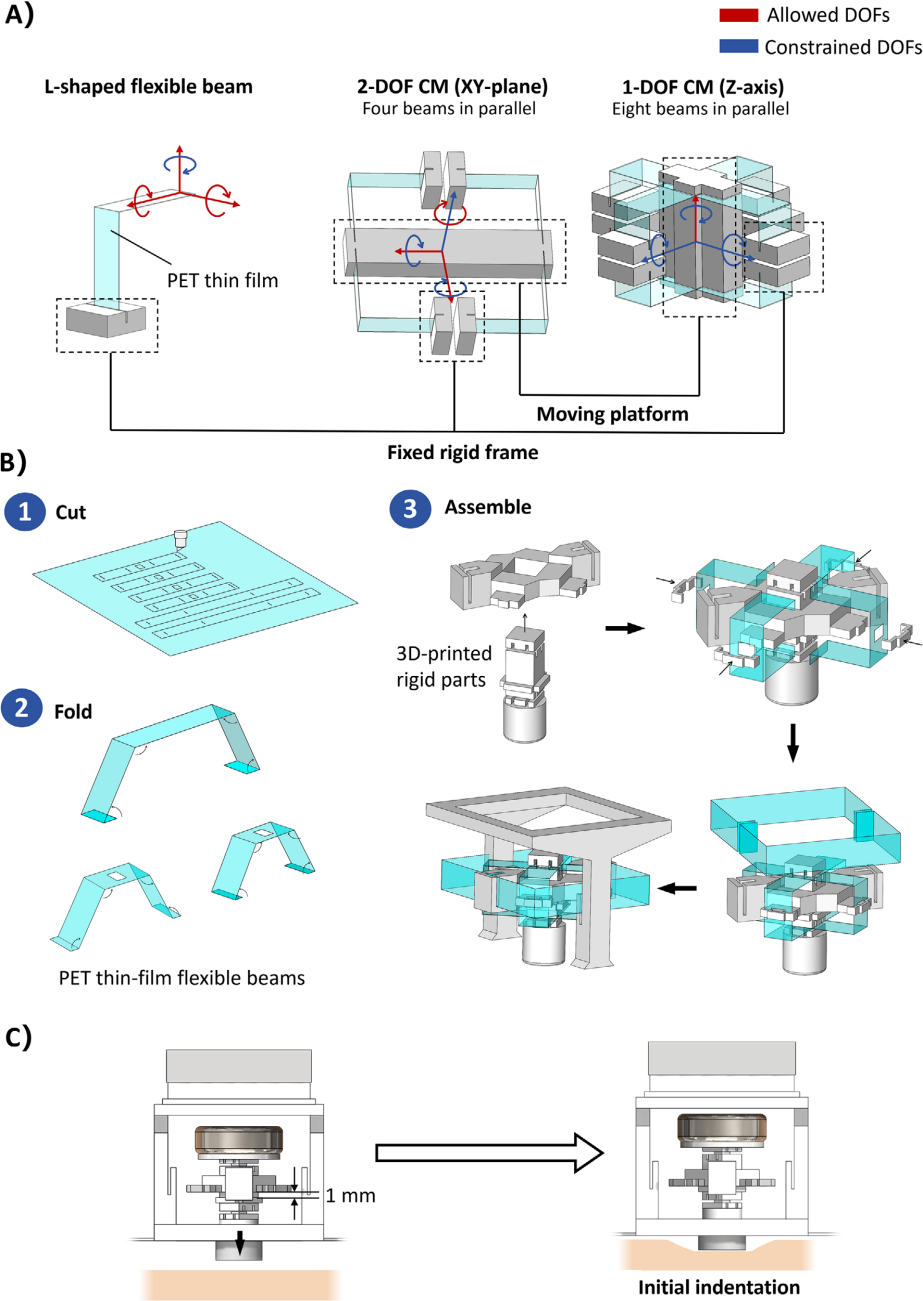
Overall structure and fabrication method of the TFCM. (A) Two compliant mechanisms constructed from PET thin‐film L‐shaped flexible beams and their degree of freedom. (B) Fabrication process of the polymer thin‐film compliant mechanism: (1) The flexible beams were cut using a cutting machine from a sheet of PET film. (2) Flexible beams were folded in the prescribed direction following the cut creases. (3) Flexible beams were inserted into the 3D‐printed rigid parts, and all the joints were bonded using a high‐strength adhesive. (C) A constraint structure designed to provide a stable initial indentation force and to constrain the spacing between the coil and the permanent magnet.

L‐shaped PET thin‐film flexible beams are the essential structural units of the TFCM. As the PET thin film exhibits high in‐plane tensile stiffness and low out‐of‐plane bending stiffness, L‐shaped flexible beams made from PET film can exhibit large deflection. Furthermore, when multiple L‐shaped flexible beams are connected in parallel, they effectively constrain unwanted degrees of freedom. In this mechanical configuration, “parallel” refers to a condition in which all L‐shaped flexible beams are connected between the same fixed frame and a moving platform (tactor in the haptic module), with one end of each beam attached to the fixed frame and the other end attached to the moving platform. Under this configuration, the beam ends connected to the moving platform undergo identical boundary displacements, and the beams collectively share the applied load. The 2‐DOF compliant mechanism (XY‐plane) consists of four L‐shaped flexible beams connected in parallel, and the 1‐DOF compliant mechanism (*Z*‐axis) consists of eight L‐shaped flexible beams connected in parallel (Figure 2A).

Figure 2B illustrates the fabrication process of the TFCM. First, a cutting machine was used to cut six PET flexible beams from the PET sheet. Next, the PET flexible beams were removed and folded along the cut creases, with each beam consisting of two L‐shaped units. Next, four flexible beams for *Z*‐axis motion and two flexible beams for *X*‐axis and *Y*‐axis motion were inserted into the corresponding 3D‐printed rigid components. Finally, they were bonded in place using the high‐strength adhesive. When haptic devices generate shear forces on the skin, a minimal lateral slip between the tactor and the skin is required to achieve effective skin stretch. To increase the maximum static friction between the tactor and the skin, we also integrated a silicone cup on the tactor. Figure 2C shows a constraint structure designed to limit the upward displacement of the tactor to within 1 mm along the *Z*‐axis. This design ensures that a sufficient initial normal indentation force is generated between the skin and the tactor to enhance friction, while also avoiding direct contact between the permanent magnet and the electromagnetic coils.

### 2.2 Force‐Displacement Characterization of the TFCM

To analyze the mechanical behaviors of the TFCM, we characterized the force‐displacement responses of both the 2‐DOF compliant mechanism (XY‐plane) and the 1‐DOF compliant mechanism (*Z*‐axis) using mathematical modelling, finite‐element simulation, and quasi‐static motion experiments. In addition, we also explored the effects of different materials, film thicknesses, and flexible beam widths on the stiffness of the TFCM in Figure S1.

**FIGURE 3:**
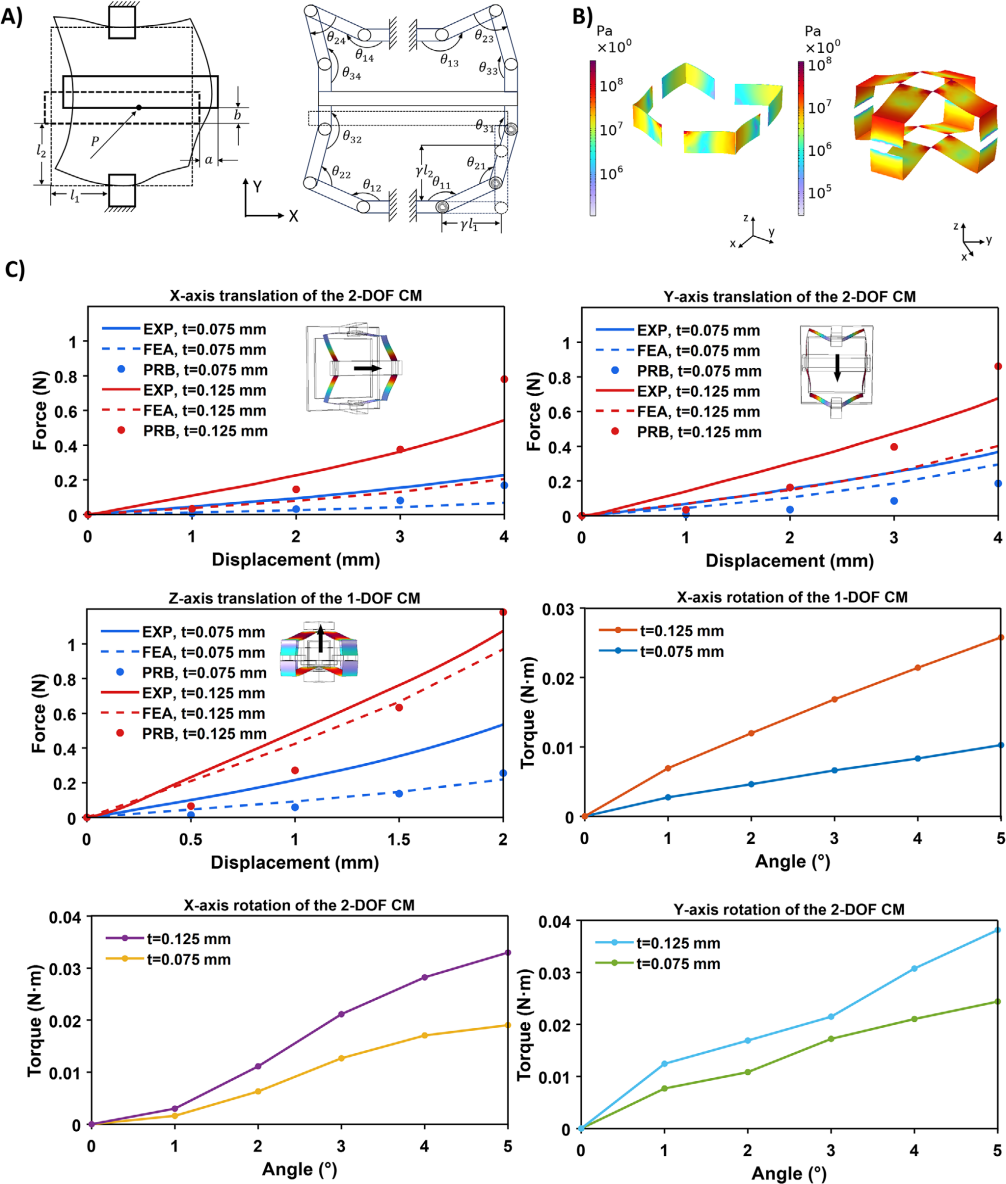
Quasi‐static force‐displacement characterization of TFCM. (A) Mathematical model of the 2‐DOF compliant mechanism using the pseudo‐rigid‐body (PRB) method. (B) COMSOL finite‐element analysis (FEA) of TFCM. (C) Force‐displacement characterization results of compliant mechanisms fabricated from PET films with thicknesses of 0.075 mm and 0.125 mm.

In the mathematical modelling part, we used the pseudo‐rigid body (PRB) method to analyze the force‐displacement relationship of a 2‐DOF planar compliant mechanism composed of four L‐shaped flexible beams. Each L‐shaped beam is discretized into an equivalent mechanism consisting of four rigid links and three revolute joints (Figure 3A). In the L‐shaped beam, the length along the *X*‐axis is *l*_1_, and the length along the *Y*‐axis is *l*_2_. And in the equivalent rigid‐body model: The length of the first rigid link is (1 − γ)*l*_1_, the second rigid link has a length of γ*l*_1_, the third link has a length of γ*l*_2_, and the fourth link has a length of (1 − γ)*l*_2_. The scale factor γ is the characteristic length coefficient in the PRB method, which is used to map the bending center of a real compliant beam to an equivalent rigid‐body rotational center, and it typically takes a value of 0.85. The three joint angles of the revolute joints are θ_1*j*_, θ_2*j*_, θ_3*j*_, where *j* = 1,  2,  3,  4.

The three joint angles satisfy the following geometric relationship:

(1) $$\begin{array}{c} \theta_{3j}=\frac{5\pi }{2}\;-\left(\theta_{1j}+\theta_{2j}\right) \end{array}$$

We can then get the geometric relationship between the joint angles and the end‐effector displacement vector (*a*, *b*):

(2) $$a={\left(-1\right)}^{j}\left[\gamma l_{1}\cos\theta_{1j}+\gamma l_{1}-\gamma l_{2}\cos\left(\theta_{2j}\right)\right]$$

(3) $$b={\left(-1\right)}^{\left\lfloor \frac{j-1}{2}\right\rfloor }\left[\gamma l_{1}\sin\theta_{1j}-\gamma l_{2}-\gamma l_{2}\sin\left(\theta_{2j}\right)\right]$$

Given a target displacement (*a_target_*,*b_target_*), an optimization problem is formulated to find the joint angles θ_1*j*_, θ_2*j*_, θ_3*j*_ that minimize the squared error between the predicted displacement and the desired one. The objective function *Objective*(θ) is defined as:

(4) $$\begin{array}{c} Objective\left(\theta \right)=\sum_{j=1}^{4}\left[{\left(a_{j}\left(\theta \right)-a_{target}\right)}^{2}+{\left(b_{j}\left(\theta \right)-b_{target}\right)}^{2}\right] \end{array}$$

Combining Equations (2) and (3), we use sequential quadratic programming to obtain the inverse solution for all joint angles. Based on joint angles, the equivalent joint torques at the revolute joints in the PRB model are computed, where *E* is the Young's modulus of the material, *I* is the area moment of inertia, and *K*_1_ and *K*_2_ are the empirical coefficients.

(5) $$\tau_{1j}=\left|\theta_{1j}-\pi \right|K_{1}\frac{EI}{l_{1}}$$

(6) $$\tau_{2j}=\left|\frac{\pi }{2}-\theta_{2j}\right|K_{2}EI$$

(7) $$\tau_{3j}=\left|\theta_{3j}-\pi \right|K_{1}\frac{EI}{l_{2}}$$

(8) $$\begin{array}{c} \tau_{j}={\left[\begin{array}{ccc} \tau_{1j} & \tau_{2j} & \tau_{3j} \end{array}\right]}^{T} \end{array}$$

According to the principle of virtual work, the reaction force *F* of the compliant mechanism can be calculated using the geometric Jacobian matrix *Q_j_* and the equivalent joint torque vector τ_*j*_ in Equation (8):

(9) $$Q_{j}=\frac{\partial \left(a,b\right)}{\partial \left(\theta_{1j},\theta_{2j},\theta_{3j}\right)}$$

(10) $$F=\sum {Q_{j}}^{-T}\tau_{j}$$

For the finite element analysis (FEA) simulation, the flexible beams were modelled using the shell feature in the COMSOL solid mechanics interface (static analysis). A prescribed displacement was applied on the compliant mechanism, and the corresponding reaction forces were extracted through surface integration. Figure 3B shows the von Mises stress distribution of TFCM during deformation in the COMSOL FEA simulation.

In the quasi‐static motion characterization experiments (Figure 3C), we obtained the force‐displacement curves of the 2‐DOF compliant mechanism along the X‐axis and Y‐axis and of the 1‐DOF compliant mechanism along the *Z*‐axis using PET films with thicknesses of 0.075 and 0.125 mm. The experimental results show that the compliant mechanism constructed with PET films of 0.125 mm in thickness exhibits approximately twice the stiffness of the mechanism constructed with 0.075 mm PET films in all directions. Due to the dimensions and the number of the L‐shaped flexible beams, the stiffness along the *Z*‐axis is significantly higher than that along the *X*‐axis and *Y*‐axis. Compared with the FEA and the PRB model, the force‐displacement curves obtained from the experiments exhibit higher linearity. In addition, we also characterized the torque produced by the two compliant mechanisms under out‐of‐plane torsional rotations ranging from 1° to 5°. The results show that peak torques at 5° exceed 0.01 Nm. This implies that when the distance between the compliant mechanism's central plane and the skin is 1 cm, a shear force of no less than 1 N would be required at the tactor tip to achieve a 5° out‐of‐plane torsional rotation. Such a shear force substantially exceeds the output capability of our device.

To achieve the desired performance, the final TFCM should exhibit high compliance in the translational directions (*X*, *Y*, and *Z*) while maintaining high stiffness in rotations about the *X*‐ and *Y*‐axes. Based on the experimental analysis, the PET film with a thickness of 0.075 mm was selected for the 1‐DOF compliant mechanism, whereas the one with 0.125 mm in thickness was used for the 2‐DOF compliant mechanism.

To evaluate the durability and mechanical reliability of the TFCM, cyclic loading tests were conducted on the structure. In the translational degrees of freedom, a displacement of 4 mm was applied, whereas in the rotational degrees of freedom, a rotational angle of 5° was applied, and the corresponding reaction forces and reaction torques of the TFCM were recorded. All the cyclic loading tests were performed for 10 000 cycles at a frequency of 2 Hz. As shown in Figure S2, the experimental results show that the stiffness of the TFCM exhibits no obvious degradation in the translational degrees of freedom, indicating that the PET thin‐film beams do not undergo significant plastic deformation or fatigue failure during translational motion. In contrast, obvious stiffness degradation is observed in the rotational degrees of freedom. In particular, the torsional stiffness about the *X*‐axis of the 2‐DOF compliant mechanism decreases by approximately 25% after 10 000 loading cycles. This behavior is likely because torsional loading induces larger localized bending and peeling stresses near the adhesive interfaces and the corners of the compliant beams, which gradually reduce the effective torsional stiffness during cyclic operation.

### 2.3 Blocked Force Characterization of the Electromagnetic Actuator

**FIGURE 4:**
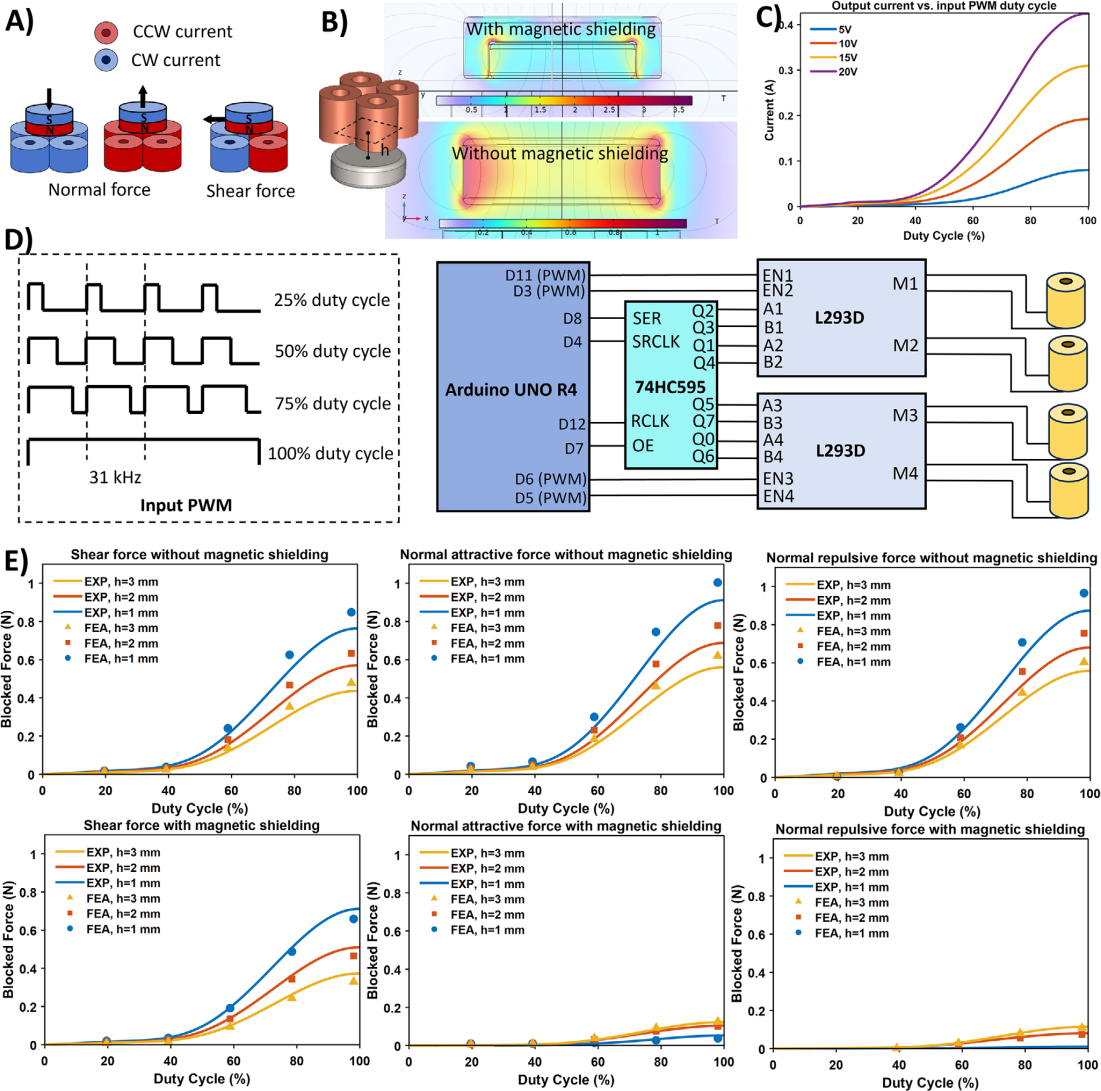
Characterization of the blocked force generated by the electromagnetic actuator. (A) Schematic of the relationship between the force acting on the permanent magnet and the current direction in the electromagnetic coils. (B) Structure of the electromagnetic actuator and magnetic field distributions of the two permanent magnets in COMSOL. (C) The relationship between the current flowing through the coil and the PWM duty cycle generated by the microcontroller. (D) Circuit schematic of the electromagnetic actuator and the PWM signals with different duty cycles. (E) Relationship between the blocked force in different directions and the PWM duty cycle generated by the microcontroller.

The electromagnetic force acting on the permanent magnet is determined by both the magnitude and direction of electric currents applied to the four electromagnetic coils and the distance between the magnet and the coils. Briefly, when the permanent magnet is centered above the coil array, the device generates specific force vectors by modulating the current direction within each coil (Figure 4A). Assume that the magnitude of the electric current applied to the four coils is equal. Uniform clockwise (CW) currents across all four coils produce a maximum normal attractive force, while reversing these to counterclockwise (CCW) currents generates a maximum repulsive force. Lateral shear force is achieved by driving the left‐right coil pair with opposing currents while maintaining identical current directions in the front‐back pair. This coordinated control of individual coil polarities allows the system to transition seamlessly between vertical displacement and horizontal translation. The detailed theoretical analysis of the static Lorentz force is shown in Note S1.

When these individual haptic modules are integrated into an array, magnetic interaction between adjacent permanent magnets creates coupling forces that influence the resulting motion of each magnet. To mitigate this interference, each permanent magnet was encased in a steel magnetic shell, effectively reducing the magnetic coupling between adjacent units. Figure 4B illustrates the magnetic flux distribution of axially magnetized N52 permanent magnets in the COMSOL model, both without and with the steel shielding shell, respectively. As shown, the steel shell changes the magnetic flux path and significantly reduces the magnetic field on the sides and top of the magnet.

To simplify the electromagnetic actuator circuit, we used a pulse width modulation (PWM)‐based DC motor control method to regulate the coil current. The PWM duty cycle determines the average voltage applied to the coils and thereby modulates the current magnitude. As shown in Figure 4C, the relationship between the average coil current and the PWM duty cycle is nonlinear: the coil current exhibits a negligible change below a 40% duty cycle with a sharp increase between 40% and 90% and a minimal variation from 90% to 100%. This relationship allows the precise control of coil current by adjusting the PWM duty cycle. The circuit implementation is illustrated in Figure 4D. A microcontroller utilizes four digital pins to interface with a 74HC595 shift register, which expands the output to eight digital signals. These signals drive the H‐bridge inputs of two L293D motor drivers, enabling precise control over the current direction in each coil. Four PWM pins (31 kHz) of the microcontroller are connected directly to the enabled pins of the two L293D microchips, where the PWM duty cycle modulates the magnitude of the coil currents.

In order to characterize the relationship between the blocked force and the PWM duty cycle in different directions, we maintained the position of permanent magnet and applied identical PWM signals (with the L293D motor driver powered by a supply voltage of 20 V) with a slow increase in duty cycles to four coils. Figure 4E shows that the blocked force increases with the PWM duty cycle in all directions. In addition, blocked‐force curves follow a trend similar to the relationship between coil current and PWM duty cycle. In the lateral direction, magnetic shielding has a slight influence on the electromagnetic force acting on the permanent magnet, and the shear force increases as the magnet‐coil distance decreases under both shielded and unshielded conditions. In the normal direction, magnetic shielding significantly reduces the electromagnetic force. Without shielding, the normal force increases as the magnet‐coil distance decreases. In contrast, with magnetic shielding, the trend is reversed, and the normal force decreases with a reduced distance. And based on the blocked force‐duty cycle curves and the coil current‐duty cycle curves, we calculated the force‐to‐power efficiency of the electromagnetic actuator ($\eta =\frac{F}{4I^{2}R}$) as shown in Figure S4. The results show that the force‐to‐power efficiency of the electromagnetic actuator decreases with increasing input current because the electrical power consumption increases quadratically with current, whereas the blocked force increases more slowly than the power consumption. To further analyze the observed force behaviors, we constructed a 3D static magnetic FEA model in COMSOL. The FEA model enables visualization of the magnetic flux distribution, which is difficult to measure experimentally in our compact configuration. Moreover, through parametric sweeps of the FEA model, we can predict both the magnitude and direction of the electromagnetic force and understand how the coil‐magnet distance and magnetic shielding influence the electromagnetic force.

In addition to the mechanical performance, we also measured the surface temperature change of the electromagnetic actuator when all four coils were driven simultaneously with identical current magnitudes, as shown in Figure S5.

### 2.4 Quasi‐Static and Dynamic Characterization of the Device

**FIGURE 5:**
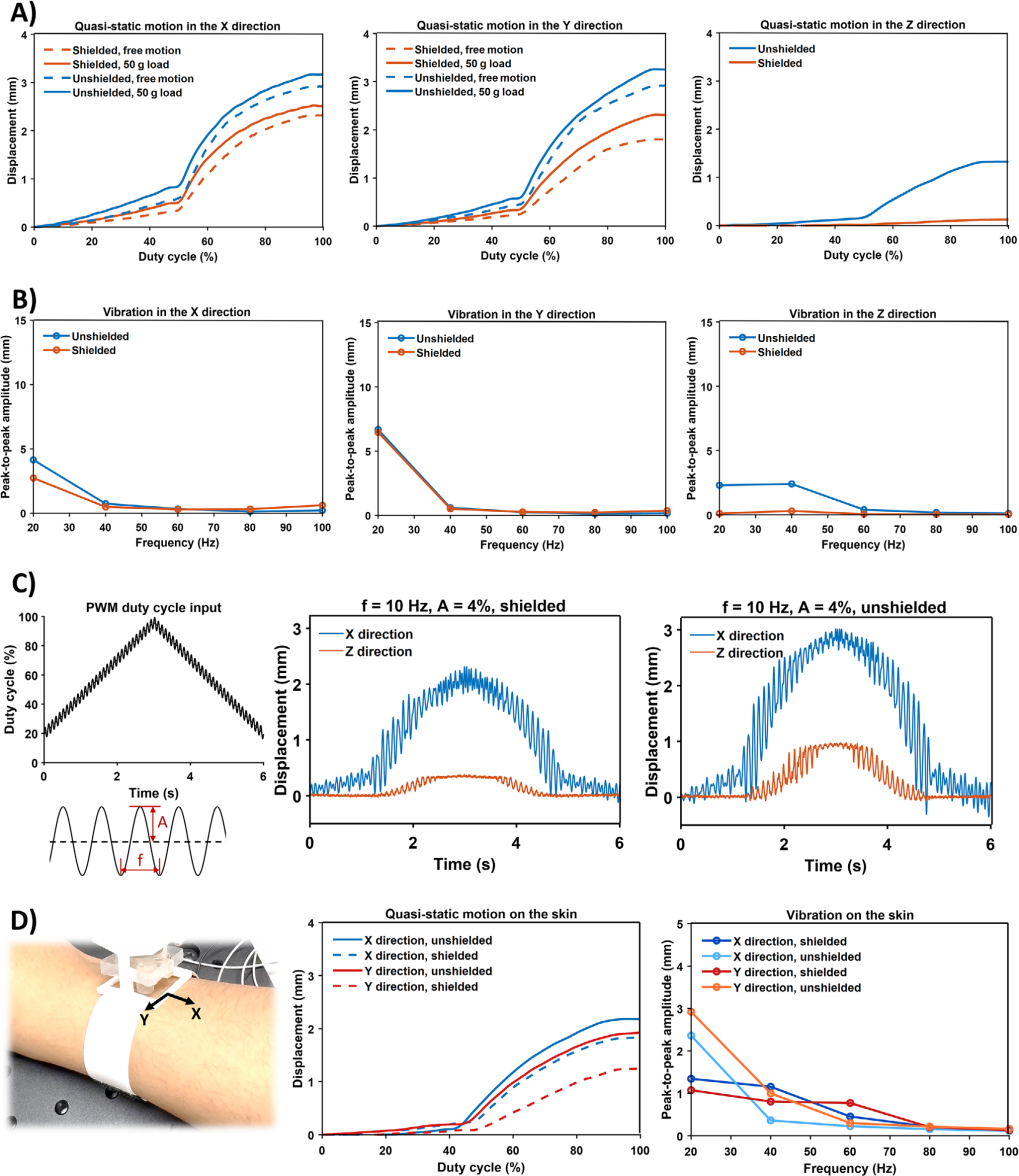
Motion characterization of the multimodal haptic device. (A) Relationship between the quasi‐static displacement of the haptic device and the input PWM duty cycle. (B) The vibration peak‐to‐peak amplitude of the haptic device measured from 20 to 100 Hz at 5 V (100% PWM duty cycle). (C) Displacement output of the device under combined vibration and shear‐force actuation. (D) Quasi‐static displacement and vibration peak‐to‐peak amplitude measured on real human skin.

Figure 5A shows the quasi‐static free motion displacement of the haptic device with a 20 V input. Because the displacement of the haptic device relies on the Lorentz force generated by the electromagnetic actuator and the elastic restoring force of TFCM, the displacement curves exhibit more complex behavior. Movie S2 shows the free motion of the device. When the duty cycle increases from 0% to 50%, there is a slow increase in the movement of the tactor. When the duty cycle exceeds 50%, the displacement increases sharply and then approaches a plateau at higher duty cycles. In all directions, the unshielded permanent magnet exhibits a larger displacement than the shielded one. This difference is particularly evident along the *Z*‐axis. Since the Lorentz force acting on the shielded magnet near the center of the coil array is nearly zero, this results in a negligible displacement from this direction. To eliminate the influence of *Z*‐axis displacement on the shear motion along the *X*‐ and *Y*‐directions, a 50 g weight was also placed on the device tactor to maintain a constant gap between the permanent magnet and the coil array. Based on the characterization of the stiffness of the TFCM in the *Z*‐direction, an external force of approximately 0.25 N is required to produce a 1 mm displacement along the Z axis. Given that the constraint structure in Figure 2C limits the maximum approach to 1 mm, a load greater than 25 g is sufficient to maintain a constant gap. In addition, we further evaluated the influence of different applied loads on both the quasi‐static displacement output and the dynamic response of the haptic module in Figure S9. In general, increasing the applied load leads to a slight increase in the quasi‐static displacement output, whereas the vibration amplitude decreases at a given frequency in the dynamic response.

The dynamic performance of the device was also evaluated in free motion under a 5 V supply at vibration frequencies of 20, 40, 60, 80, and 100 Hz, respectively (Figure 5B). Movie S3 shows the vibration of the device. In all directions, the peak‐to‐peak vibration amplitude decreases as the vibration frequency increases. As shown in Note S2, this behavior is attributed to the inertia of the permanent magnet and the damping of the passive components (see Supporting Information). Reducing the mass of the permanent magnet and the system damping can improve the high‐frequency amplitude.

In addition, the capability of the device to simultaneously generate shear force and vibration was also evaluated (Figure 5C). An X‐direction shear‐force input with a superimposed vibration was applied to the electromagnetic actuator. The PWM duty cycle used to generate the pure shear force followed a triangular waveform, rising linearly from 20% to 96% during the first 3 s and then decreasing linearly back to 20% over the subsequent 3 s. The superimposed vibration component was a sinusoidal duty‐cycle modulation with amplitude A and frequency f. By setting A = 4% and f = 10 Hz, we measured the device's displacement in the X and Z directions in free motion under 20 V. The results show significant displacement in both shear and normal directions. Furthermore, as the permanent magnet moves away from the center of the coil array, the vibration amplitude in both directions first increases and then decreases.

Figure 5D shows the quasi‐static displacement and peak‐to‐peak vibration amplitude in the shear direction on hairy forearm skin, while Figure S10 shows the corresponding results in the normal direction without magnetic shielding (see Supporting Information). Compared with the free‐motion condition, all the displacement and vibration amplitude of the device are reduced. Movie S4 shows the motion of the haptic device on hairy forearm skin. We also used the Tracker software to measure the parasitic torsional angle of the permanent magnet during shear motion on the skin (Figure S12). The measured torsional angle was approximately 1.6° during shear motion along the *Y*‐axis and approximately 2.2° during shear motion along the *X*‐axis. These results demonstrate that the TFCM effectively constrains unwanted DOFs during shear motion.

To evaluate the long‐term operational stability of the haptic device, the actuator was continuously operated under free‐motion shear actuation for 40 min at a frequency of 5 Hz with an amplitude of approximately 3 mm, while the displacement output was recorded throughout the experiment. As shown in Figure S11, the experimental results show that the device exhibits no obvious degradation in displacement output during prolonged large‐amplitude operation.

### 2.5 Trajectory Control

**FIGURE 6:**
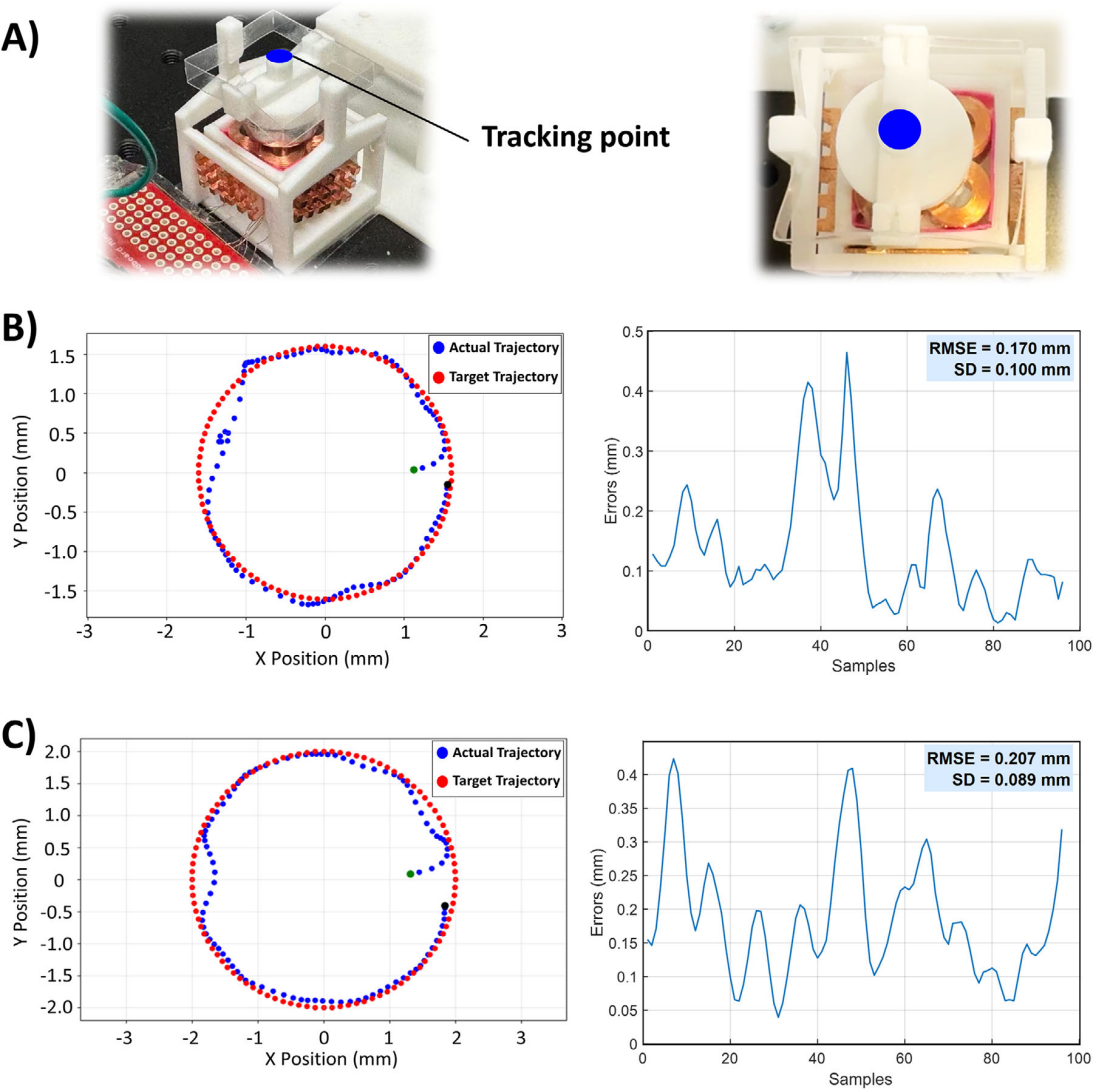
Trajectory control of the permanent magnet in the planar 2‐DOF compliant mechanism. (A) Experimental setup and tracking points for trajectory control. (B) Circular trajectory tracking with a radius of 1.6 mm. (C) Circular trajectory tracking with a radius of 2 mm.

Real‐time and accurate regulation of the electric currents in the four coils can enable control of the tactor's motion trajectory. We validated the feasibility of planar trajectory control on the XY‐plane 2‐DOF compliant mechanism based on a vision‐language MPC framework (Figure 6A). The control system of the device allows for drawing any arbitrary trajectory within the workspace. Starting from the PPS‐VLMPC framework, we dropped the VLM module using the MPC with the learned dynamics and assigning the trajectory with standard trajectory planning methods. In our case, the neural network learned the end‐to‐end forward kinematics, mapping directly from the PWM duty cycle of each coil $u\in R^{2}$ to the Cartesian coordinates of the tactor $p=\left(x,y\right)\in R^{2}$. The latter was used as the state function of the MPC, allowing the computation of the control input that forces the device to follow desired reference trajectories. For the circular trajectory with a radius of 1.6 mm in Figure 6B, the controller achieves an RMSE of 0.170 mm with a standard deviation of 0.100 mm. For the circular trajectory with a radius of 2 mm in Figure 6C, the RMSE increases slightly to 0.207 mm, while the standard deviation is 0.089 mm. From these results, our controller shows high tracking accuracy and stable performance. And Movie S6 demonstrates the dynamic process of repeatedly generating circular trajectories with a radius of 2 mm.

This trajectory control capability may enable future dynamic tactile displays (or haptic textual language) to assist visually impaired users. The haptic device can continuously render arbitrary geometric patterns directly on the skin, such as circles, squares, letters, and numbers. Compared with simple directional shear and vibration cues, continuous geometric trajectory rendering theoretically provides a higher‐bandwidth tactile communication channel. In addition to haptic applications, our device may also be applied to the wearable skin‐surface 3D printing system. By precisely controlling the trajectory of a miniature 3D‐printing nozzle, the system could enable customizable deposition of functional materials, such as liquid metals and hydrogels, for applications including physiological monitoring through 3D printing of flexible electronics and localized transdermal drug delivery.

### 2.6 User Study

**FIGURE 7:**
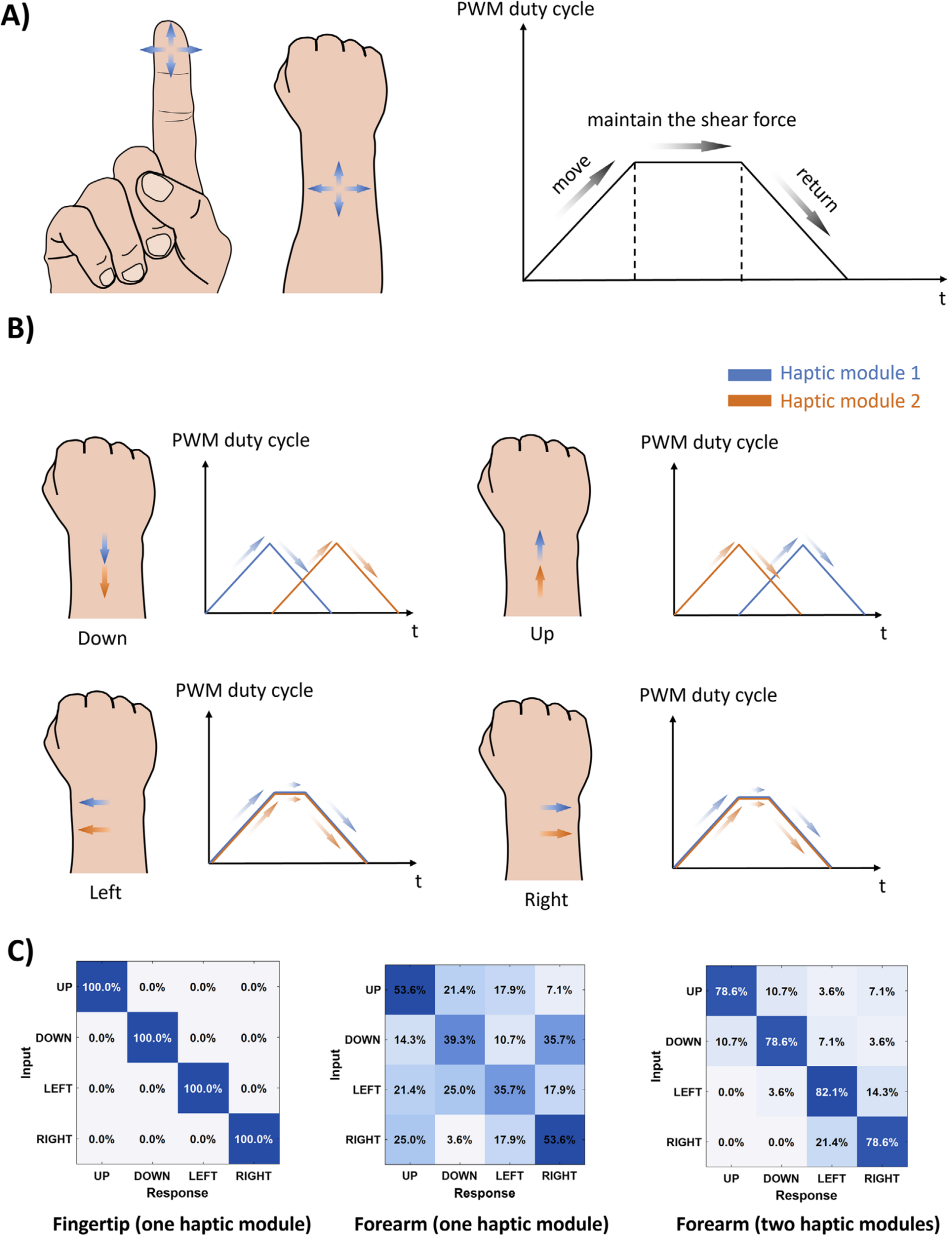
User study on four‐direction discrimination of shear‐force cues. (A) Directional signal encoding strategy of the single haptic module on finger and forearm skin. (B) Spatiotemporal encoding strategy using two haptic modules on the forearm skin. (C) Confusion matrix of the user study results.

To demonstrate the effectiveness of our system, we also conducted a series of user studies with human subjects (N = 7) under the UNSW Ethics approval number HC210451 (see Experimental Section). In this study, we first analyzed how different skin locations and haptic encoding strategies influence users’ accuracy in perceiving shear forces in all four directions. Figure 7A illustrates the encoding strategy for directional cues delivered by a single haptic module on the skin of the right index finger and the right forearm. The duty cycle of the four sets of PWM waves which control the four electromagnetic coils remained identical, and the direction of the coil current was determined by the target direction of the tactile signal. The input duty cycle first increased linearly to move the tactor toward the target direction. It then remained at 100% to maintain a constant shear force on the skin, providing users with sufficient time to identify the direction. Finally, the duty cycle decreased linearly, returning the tactor to its initial position. In addition, we also explored the use of two haptic modules and a spatiotemporal encoding strategy to improve users’ accuracy in discriminating among four directions on the forearm. Two haptic modules were arranged longitudinally along the forearm with a tactor‐to‐tactor spacing of 35 mm. We then set one module closer to the hand (module 1) and the other further from the hand (module 2). As shown in Figure 7B, when delivering a DOWN cue, both modules’ tactors generated downward shear forces, but haptic module 1 moved first, followed by haptic module 2. When delivering an UP cue, both modules’ tactors generated upward shear forces, but the haptic module 2 moved first, followed by module 1. For the LEFT and RIGHT cues, the two modules always moved simultaneously in the same direction. The spatiotemporal encoding strategy enabled users to clearly distinguish the UP and DOWN cues based on the temporal sequence of skin stretch stimulation. For the LEFT and RIGHT directions, the two tactors increased the effective area of tactile stimulation to improve users’ discrimination accuracy.

Figure 7C shows all the results of the user study. On the glabrous skin of the fingertip, all users were able to accurately identify the four‐direction shear cues produced by a single haptic module, achieving an accuracy of 100%. However, the density of mechanoreceptors on the forearm is less than 5% of that on the finger, making directional cue delivery more challenging [26]. When using a single haptic module to deliver four‐direction shear cues on the hairy skin of the forearm, only the UP and RIGHT cues achieved accuracy above 50%, and the overall mean accuracy was only 45.54%. The data suggests that users can hardly perceive four‐direction shear cues generated by a single tactor on the forearm (with an effective skin‐stretch displacement smaller than 2 mm). This result is consistent with previous studies: Kyled et al. reported that a large skin‐stretch displacement (14.3 mm) enabled users to achieve an accuracy of 85.1% for four directions, whereas the accuracy dropped to 43.5% for eight directions [36]. When two multimodal haptic modules were used on the forearm together with the spatiotemporal encoding strategy, the mean recognition accuracy increased to 79.5%, which was nearly double that achieved with a single haptic module. Most users could clearly distinguish the UP‐DOWN and LEFT‐RIGHT directional pairs but could not achieve 100% accuracy in discriminating the two individual cues within each pair. The result suggests that a haptic array composed of multiple multimodal modules, combined with a spatiotemporal encoding strategy, can substantially improve the perception accuracy of complex directional cues. Without the spatiotemporal encoding strategy, a substantially larger multimodal haptic module with higher power consumption would be required to maintain high directional discrimination accuracy on the forearm. Conversely, if the spatiotemporal encoding strategy were used with only single‐DOF haptic modules, at least four separate haptic modules would be required to achieve reliable directional perception. Therefore, spatiotemporal encoding and multimodal haptic feedback are mutually dependent components that together reduce the number of required haptic modules as well as the overall size and power consumption of wearable haptic arrays.

In addition to the confusion matrix results of four‐direction discrimination, one user achieved 100% discrimination accuracy on the forearm when using two multimodal haptic modules and reached 81.3% accuracy with a single module after extensive haptic training. This finding highlights the potential of haptic training to improve users’ ability to perceive haptic signals.

**FIGURE 8:**
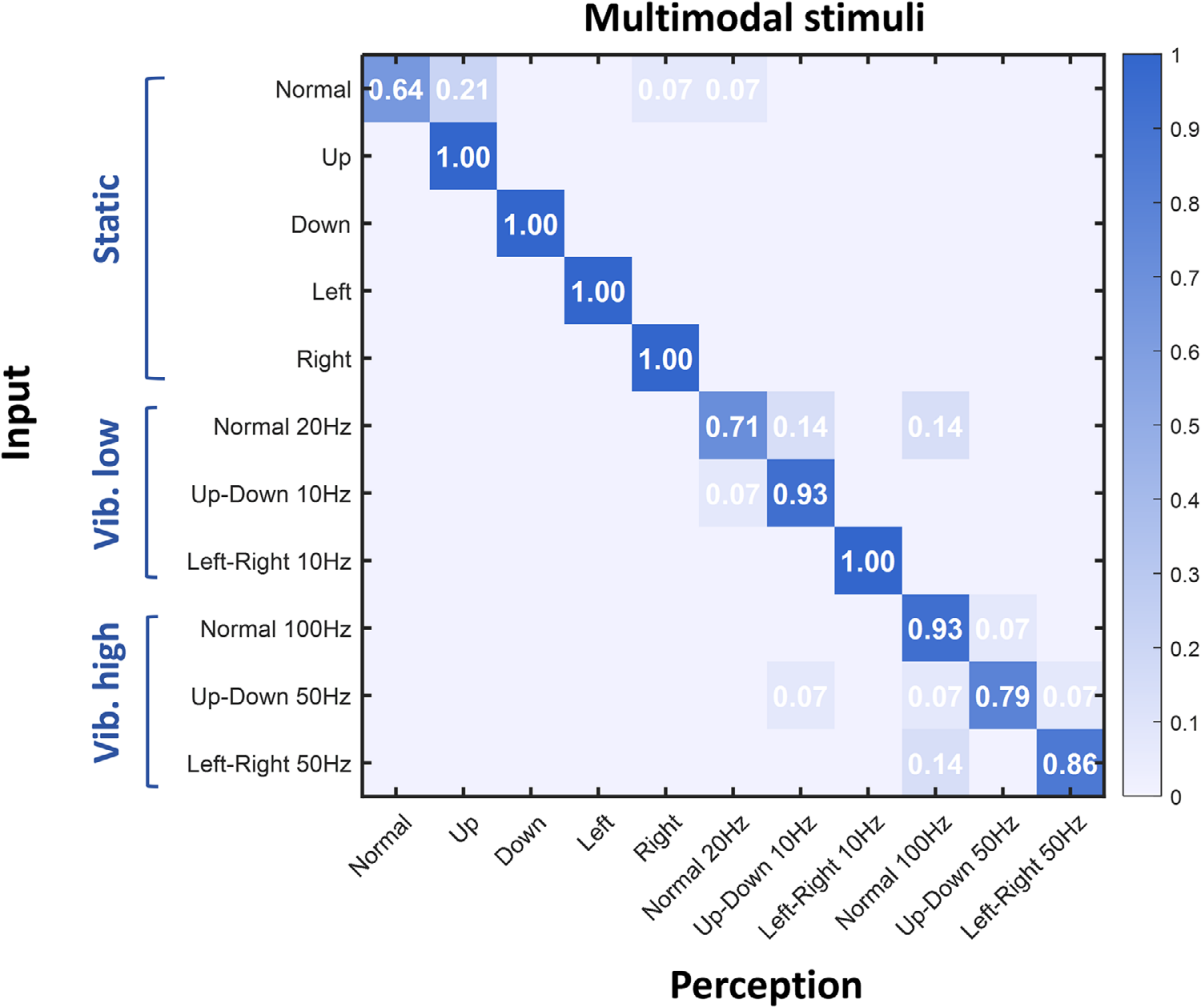
User study on discriminating 11 mechanical stimuli on the fingertip. These stimuli cover normal indentation, skin stretch, and vibration.

To further validate the multimodal haptic rendering capability of the proposed device, we conducted a multimodal stimulus perception user study. In this experiment, 11 different tactile stimuli were delivered to the users’ fingertips, including five static directional force stimuli (UP, DOWN, LEFT, RIGHT, and NORMAL), three high‐frequency vibration stimuli with different directions, and three low‐frequency vibration stimuli with different directions. These stimuli cover normal indentation, skin stretch, and vibration feedback. Figure 8 presents the corresponding confusion matrix of the user study. The result shows that users achieved an overall discrimination accuracy of 89.6% across all 11 stimuli, demonstrating that the proposed device can render distinct multimodal haptic feedback.

Besides the two user studies above, we also characterized the perceptual thresholds under different stimulation modes (normal, north‐south shear, east‐west shear) and vibration frequencies (static, 25 Hz, 50 Hz, 75 Hz, and 100 Hz) on both the fingertip and forearm skin (see Supporting Information). The perceptual thresholds were quantified using the input current of the haptic device. As shown in Figure S13, the experimental results demonstrate two main conclusions: (1) the perceptual thresholds on the forearm are significantly higher than those on the fingertip; and (2) except for the static normal stimulus, all perceptual threshold currents remained below 40 mA on the fingertip and below 80 mA on the forearm.

### 2.7 Proof‐of‐Concept Demo

**FIGURE 9:**
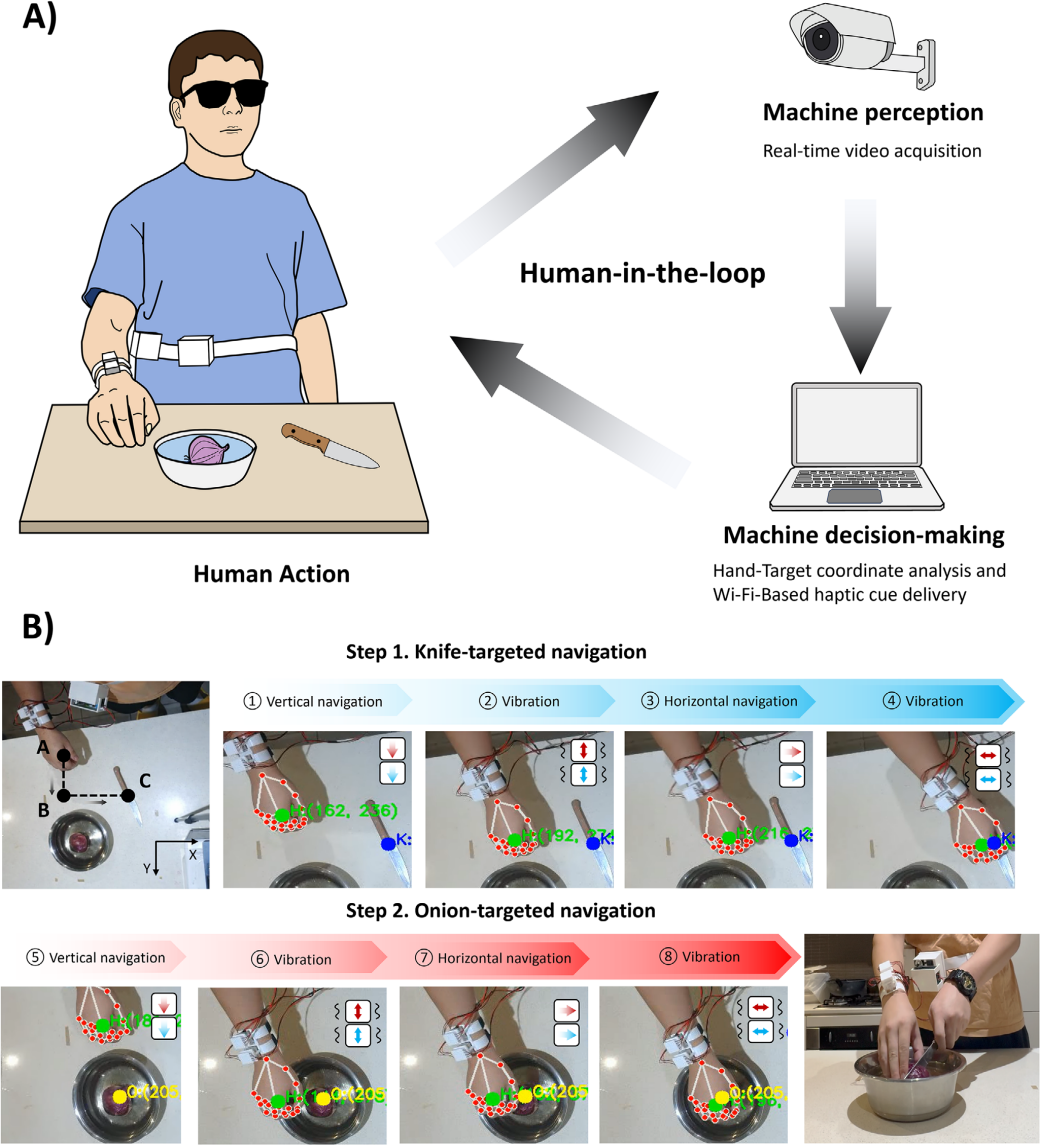
Hand navigation for the user with eliminated vision using cutaneous haptic cues. (A) Schematic of a closed‐loop hand navigation system based on the Wi‐Fi‐controlled haptic device. (B) Sequential images illustrating the hand being guided toward the knife and onion, including the haptic cues delivered at different steps.

This section presents a proof‐of‐concept demonstration of our haptic device to assist visually impaired users in the future when performing daily activities by delivering directional haptic cues to the forearm. In this paper, the demonstration was conducted with a single user (healthy participant with eyes closed) and was designed to validate the feasibility of the proposed technology. In future work, we will introduce more complex task scenarios and conduct experiments with a larger number of users. As shown in Figure 9A, we developed a vision‐guided human‐in‐the‐loop (HITL) haptic assistance system to support users during food preparation. This system consists of three stages: (i) machine perception, (ii) machine decision‐making, and (iii) human action. During the machine perception stage, a camera continuously captures video of the workspace throughout the task. In the machine decision‐making stage, a YOLO‐based object detection pipeline is employed to estimate the relative positions of the user's hand and the target object in real time. The computed positional differences are then converted into directional haptic cues and delivered through Wi‐Fi to the haptic device (Figure S14). In the human action stage, the user perceives the haptic cues and moves their hand toward the target object.

Based on the closed‐loop system, we constructed a task scenario designed to assist visually impaired users in cutting an onion (Movie S5). The experiment was performed with a sighted user who simulated visual impairment by closing his eyes during the task. As the target objects were placed on the same tabletop plane, planar directional haptic cues were sufficient to guide the user toward grasping the object. Therefore, in this study, we only used a 2D planar coordinate system. The user could move the hand to the planar target location and then lower the hand to grasp the object. Future work will integrate 3D directional cues to support more complex task scenarios.

First, the user placed their right hand on the kitchen countertop and issued a voice command to the computer: ‘I want to cut an onion’. Upon receiving the command, the system began real‐time tracking of the user's right‐hand coordinates and planned a navigation path from the initial hand position (point A), through an intermediate point (point B), to the target object location (point C) to guide object grasping. All coordinates were defined in pixel units within the image coordinate system. As shown in Figure 9B, the system first aligned the vertical position of the user's hand with that of the knife, guiding the hand from point A to point B. In this stage, the knife's vertical coordinate was defined as *y_target_*, the user's hand vertical coordinate as *y_hand_*, and their difference as

$$\Delta y=y_{target}-y_{hand}$$

A threshold of 5 pixels was defined to determine whether the user's hand had reached the target location. When Δ*y* > 5, the haptic device output an UP cue, prompting the user to move the hand forward. When Δ*y* < −5, a DOWN cue was generated, guiding the user to move the hand backward. When |Δ*y*| < 5, the haptic device produced a vibration cue in the UP‐DOWN direction, indicating that vertical navigation had been completed. At this stage, the user's right hand had been guided to point B, where its vertical coordinate was aligned with that of the target object. Next, the system aligned the horizontal position of the user's hand with that of the knife (From point B to point C). The horizontal coordinates of the knife and the hand were denoted as *x_target_* and *x_hand_*, respectively, with

$$\Delta x=x_{target}-x_{hand}$$

When Δ*x* > 5, a LEFT cue was delivered, whereas a RIGHT cue was generated when Δ*x* < −5. Once |Δ*x*| < 5, the haptic device vibrated in the LEFT‐RIGHT direction, signalling the completion of horizontal navigation. After the hand was successfully guided to the knife, the system switched the target to the onion and applied the same navigation logic. Following this procedure, the user was able to complete the task of cutting the onion in water. Compared with the multimodal haptic devices worn on the fingers or hands [28, 42], our forearm‐mounted haptic device doesn't interfere with fine finger manipulation. And when the hand is immersed in water during operation, the forearm‐mounted device doesn't require any additional waterproofing. And compared with conventional forearm‐mounted haptic device, which either rely on large haptic arrays composed of single‐DOF haptic modules [47, 48] or bulky high‐force actuators without spatiotemporal encoding [36, 49, 50, 51], our design combines multimodal haptic feedback with spatiotemporal encoding, thereby reducing the number of actuators required in the haptic array and enabling a more compact overall haptic system.

## 3 Discussion and Conclusions

This work presents a multimodal electromagnetic cutaneous haptic module based on a 3‐DOF PET thin‐film compliant mechanism. The device exhibits three advantages enabled by: (1) TFCM's large‐displacement capability allows the haptic module to generate sufficient effective skin deformation, (2) TFCM's low resistance enables better transmission of the electromagnetic force to the skin, resulting in a larger output force, and (3) TFCM effectively constrains unwanted rotation during skin‐stretch.

We characterized TFCM by measuring its translational stiffness along the *X*, *Y*, and *Z* axes and its torsional stiffness about the *X* and *Y* axes, demonstrating high compliance along the wanted motion directions and high stiffness along unwanted directions. We then characterized the blocked electromagnetic forces generated by a 2 × 2 coil array acting on the permanent magnet and investigated the influence of magnetic shielding on the resulting magnetic forces. Next, the quasi‐static and dynamic displacements of the haptic module along three translational directions were measured, both in free motion and on forearm skin, and the feasibility of superimposing shear force and vibration was validated. During force and displacement characterization, we observed that magnetic shielding significantly affects the output performance, leading to a limited Z‐axis displacement (less than 0.5 mm). Under this condition, the system may not fully achieve effective 3‐DOF actuation performance.

Furthermore, we implemented planar trajectory control of the haptic module (using the 2‐DOF in‐plane compliant mechanism) based on a vision‐language MPC framework. In the user study, we conducted a four‐direction shear‐force discrimination experiment. The results showed that users could achieve 100% accuracy in identifying shear directions generated by a single haptic module on the fingertip but exhibited poor discrimination performance on hairy forearm skin (43.5% accuracy). By using two haptic modules combined with a spatiotemporal encoding strategy, the discrimination accuracy on the forearm increased to 79.5%, demonstrating that the haptic array with a spatiotemporal encoding method could enhance the accuracy. Finally, we realized Wi‐Fi‐based wireless control of the haptic device and developed a vision‐guided human‐in‐the‐loop (HITL) haptic assistance system. Using this system, an onion‐cutting task was demonstrated, which highlights the potential of our haptic device for future applications in assistive technologies for the visually impaired.

To demonstrate the innovative approach of our work, Table 1 compares existing multimodal cutaneous haptic devices (mechanical stimulation) with our device. Our device has a balanced performance: Compared with other EM actuator‐based haptic devices, it provides a larger force and displacement, enabling effective haptic signal delivery on hairy skin. In contrast to devices driven by pneumatic actuators or motors, our device is more compact, and compared with devices based on SMA actuators, our device achieves higher vibration frequencies.

**TABLE 1:** Comparison of existing multimodal haptic devices.

References	Size [mm]	Actuator	DOF	Free Motion Range [mm]	Blocking Force [N]	Max. Vibrational Frequency [HZ]	Simultaneous Actuation of Shear Force and Vibration	Tethered	Mass [g]
This work	31 × 28 × 33	EM	N, 2‐axis Sh	Sh: 3.3 N: 1.3	Sh: 0.76, N: 0.91	500	Yes	No	33
[33]	80 × 80 × 45	Motor	N, 2‐axis Sh, T	Sh: 13 N: 8	Sh: 1.5, N: 1.6	9	No	Yes	50
[16]	NR	Hydraulic	N, 2‐axis Sh	Sh: 1.5 N: 3	Sh: 1.5 N: 1.7	NR	Yes	Yes	NR
[34]	55 × 55 × 20	Pneumatic	N, 2‐axis Sh	Sh: 6 N: NR	Sh: 3.3 N: 8.3	250	Yes	Yes	25
[36]	113 × 113 × 35	Pneumatic	N, 2‐axis Sh	Sh: 7 N: NR	Sh: 0.6 N: 0.6	NR	Yes	Yes	120
[35]	40 × 20	Pneumatic	N, 2‐axis Sh, T	Sh: 3 N: 4	Sh: 1.3 N: 7	64	No	Yes	NR
[44]	6 × 6 × 0.8	Electrostatic	N, 2‐axis Sh	Sh: 0.76 N: 0.5	Sh: NR N: 0.3	200	No	No	0.09
[42]	NR	SMA	N, 2‐axis Sh	Sh: 1 N: NR	Sh: 0.85 N: NR	2<	No	No	NR
[43]	29 × 29 × 30	SMA	N, 2‐axis Sh, T	Sh: 0.6 N: NR	Sh: NR N: 12	NR	No	Yes	24.4
[45]	15 × 15 × 4	EM	N, 2‐axis Sh, T	Sh: 3 N: 1.8	Sh: 0.005 N: 0.028	200	No	Yes	0.8
[28]	8.4 × 11.7 × 10.8	EM	N, 2‐axis Sh	Sh: 0.9 N: 0.6	Sh: 0.15 N: 0.3	500	Yes	No	3.19
[46]	24 × 24 × 20	EM	N, 2‐axis Sh	Sh: 1 N: 1	Sh: NR N: 0.4	Sh: 50 N: 333	Yes	Yes	19

^a^ N: normal.

^b^ Sh: shear.

^c^ T: torsion.

^d^ NR: not reported.

Although haptic output performance and device size are important metrics, wearability should not be overlooked for skin‐mounted haptic interfaces. Adhesive‐based haptic devices can provide compact and effective actuator‐skin coupling; however, adhesive removal may cause skin irritation [52], discomfort [53], and removal‐related pain [54], and the mechanical reliability can degrade after repeated attachment‐removal cycles [18]. To evaluate the limitation, we measured the repeated 90° peeling force of two representative double‐sided tapes on the dorsal hand skin and tested the long‐term operational stability of adhesive‐mounted permanent magnets on the finger under shear vibratory stimulation. The peeling‐force experiment shows that the adhesive strength of the tapes was substantially reduced after removal, indicating degraded re‐adhesion capability (see Figure S21). In the long‐term vibration test (see Movie S7), a 15 mm‐diameter adhesive‐mounted magnet could operate for more than 10 min due to its larger contact area, but the adhesive layer had to be replaced after removal because it could no longer reliably re‐adhere to the skin. In contrast, a 6 mm‐diameter adhesive‐mounted magnet detached spontaneously after approximately 1 min of operation due to its smaller contact area. These results suggest that adhesive‐based haptic structures may require replacement of the adhesive layer after each attachment‐removal cycle and may suffer from mechanical instability during prolonged operation. By contrast, our device provides an adhesive‐free and reusable mechanical force‐transmission interface, enabling repeated and extended haptic operation without replacing skin‐contact adhesive components.

In future work, we aim to further reduce the device size and integrate a larger number of haptic modules into the array to improve the spatial resolution of haptic signals. In addition, long‐term operation of EM coils can lead to heat accumulation and performance degradation. To address this issue, future work will focus on improving both the passive and active thermal management capability of the system. First, the 3D‐printed housing of the electromagnetic actuator can be redesigned with perforated structures, and miniature copper heat‐dissipation fins can be directly bonded to the coil surfaces using thermally conductive adhesive to enhance passive heat dissipation. And active cooling approaches, such as integrating Peltier cooling modules and miniature fans into the electromagnetic actuator, may also be employed for enhanced thermal management.

To mitigate the performance degradation caused by magnetic shielding, we propose two structural optimization strategies for future work. First, the magnetic shielding shell around the permanent magnet can be removed and replaced with a shielding configuration for the entire haptic module. In this approach, a shielding shell made of high‐permeability materials, such as permalloy or soft iron sheets, is used to surround the entire haptic module. This design can preserve the internal magnetic field distribution required for actuation while reducing magnetic interference between adjacent modules. Alternatively, if the pot magnet is still used, an additional planar coil aligned with the center of the shielding structure could be integrated. Activating this coil can enhance the normal electromagnetic force along the *Z*‐axis.

In terms of applications, we plan to use the multimodal haptic array combined with spatiotemporal encoding strategies to render complex interactive behaviors in virtual environments on large skin areas.

Beyond the current electromagnetic implementation, the proposed TFCM could also serve as a general passive compliant mechanical architecture for multimodal cutaneous haptic interfaces using different actuation technologies and enable more stable and controllable multimodal tactile rendering.

## 4 Experiment Section

### 4.1 Materials

The 0.075‐mm PET sheet was purchased from an online retailer on eBay Australia, while the 0.125‐mm PET film (Mylar Plastic Film, 304 mm × 200 mm × 0.125 mm) was obtained from RS Components Australia. The axially magnetized N52 permanent magnet with a 15 mm diameter was sourced from Lalaci Magnet Company, and the 16 mm N52 pot magnet (magnetic shielding) was custom fabricated by SUNHUY Company. The electromagnetic coils used in this work (Juneng Electromechanical Co., Ltd.) had a height of 10 mm, an outer diameter of 9 mm, and an inner diameter of 4 mm. Each coil had a resistance of 26 Ω and was wound with 0.16 mm enameled copper wire for a total of approximately 1000 turns.

### 4.2 Fabrication of TFCM

A cutting machine (Cameo 4) was used to cut the PET film into two types of flexible beams (Figure S15A), whose dimensions are shown in Figure S15B. Next, all the flexible beams were folded along the predefined creases and bonded to their corresponding 3D‐printed parts using super glue (UHU). The four shorter beams formed the Z‐direction compliant mechanism and were attached to two transparent resin parts fabricated using SLA 3D printing (Formlabs Form 4), whereas the two longer beams formed the XY‐direction compliant mechanism and were bonded to a PLA part fabricated using FDM printing (Bambu Lab X1E).

### 4.3 Force‐Displacement Testing of TFCM

Figure S16 shows the four characterized compliant mechanisms (including the XY‐plane compliant mechanism and the Z‐axis compliant mechanism constructed with PET films of two different thicknesses) and the four characterization setups. For characterizing the force‐displacement behavior along the X, Y, and Z translational axes, the compliant mechanism was mounted on the moving stage of the linear actuator (Zaber), while a six‐axis force‐torque sensor (ATI Nano17) was fixed to the stationary base of the linear actuator. The motor was driven at 0.5 mm s^−1^ to bring the compliant mechanism into contact with the sensor, and the reaction forces generated by the flexible beams were recorded during the movement (0‐4 mm in the X and Y directions, and 0‐2 mm in the Z direction).

To characterize the torque‐angle relationship for rotations about the *X* and *Y* axes, a stepper motor (PKP) was mounted at the lower end of the setup and connected to the sensor through a 3D‐printed connector, with the compliant mechanism fixed above the sensor. The stepper motor was commanded to rotate in 1° increments, pausing at each step to record the corresponding torque, until a total rotation of 5° was reached.

### 4.4 Blocked Force Measurements of the Electromagnetic Actuator

We used an L203D Motor Control Shield (Duinotech) as the motor driver, an Arduino Uno Wi‐Fi R4 as the main controller, and ACS723 current sensors (SparkFun) for current measurement. The input voltage supplied to the motor driver was set to 5, 10, 15, and 20 V. The controller output a PWM signal whose duty cycle linearly increased from 0% to 100% over 0–3 s. And the outputs from the four current sensors were recorded and averaged (Figure S17).

The 2 × 2 coil array was fixed beneath the permanent magnet, while the ATI Nano17 sensor and a Z‐axis linear stage for adjusting the magnet‐coil spacing were mounted above the magnet in sequence (Figure S18). The PWM signal with a linearly varying duty cycle was again used to control the current amplitude in the four coils, and four digital pins were used to set the coil current directions. The distance between the coils and the permanent magnet was adjusted to 1, 2, and 3 mm, and the resulting magnetic forces were recorded.

### 4.5 Motion Measurements of the Haptic Device

The laser displacement sensor (KEYENCE IL‐100) was used to measure the device's displacement in different directions. As shown in Figure S19, in the free‐motion measurement experiment, a white 3D‐printed part was attached to the tactor to provide a reflective surface for the laser and to hold a 50 g weight. For the on‐skin displacement measurements, a small piece of white paper was attached to the *X*‐*Y*‐plane movable 3D‐printed part of the device to act as the reflective target. For the quasi‐static motion tests, the input voltage was set to 20 V, and the duty cycle of the PWM input increased linearly from 0% to 100% over 0–3 s. For the dynamic tests, the input voltage was set to 5 V, and the PWM duty cycle was driven at vibration frequencies swept from 20 to 100 Hz in 20 Hz increments.

### 4.6 Data Collection for Trajectory Control

To enlarge the tactor's motion range and reduce the current‐induced temperature rise during prolonged position data acquisition, we used the unshielded permanent magnet and the 0.075‐mm flexible beams in this experiment. At an input voltage of 5 V, random input trajectories were generated by applying PWM signals with randomly varying duty cycles to the motor driver. The resulting trajectories were recorded using a 13‐megapixel RGB camera (OAK‐D‐Lite) for model training.

### 4.7 User Study

The user study included seven participants (aged 20–30 years, with no dermatological or psychological conditions), and all participants were non‐experts in haptic feedback devices. The Human Research Ethics Advisory Panel (HREAP) at UNSW approved the research protocol under ethics number HC210451. As shown in Figure S20, we developed a GUI for the user study using Processing software, which included an exploration mode and a testing mode.

For the user study on four‐direction discrimination of shear‐force cues, in the exploration mode, participants could click on directional buttons on the screen to trigger the corresponding directional motion of the haptic device. Then participants proceeded to the testing mode, in which the haptic device delivered 16 directional stimuli in a randomized order (four per direction, and each stimulus was repeated twice). Upon perceiving a tactile cue, participants were required to select the corresponding direction on the screen, and the computer recorded both the randomly generated stimulus direction and the participant's response. During the entire testing procedure, participants were not allowed to observe the movement of the haptic device on their forearms.

For the user study on discriminating multimodal stimuli on the fingertip, we encoded 11 different tactile stimuli, including five‐direction static force stimuli, three low‐frequency vibration stimuli (20 Hz), and three high‐frequency vibration stimuli (100 Hz). The static force stimuli included NORMAL, UP, DOWN, LEFT, and RIGHT directions, while the vibration stimuli included normal vibration, UP‐DOWN shear vibration, and LEFT‐RIGHT shear vibration. Because normal vibration stimulates the skin once per cycle whereas shear vibration stimulates the skin twice per cycle, the frequency of normal vibration was set to be twice that of shear vibration. A PWM duty cycle of 100% was used for static force stimuli, whereas a reduced duty cycle of 68% was used for vibration stimuli because vibration could enhance the amplitude. In the exploration mode, users sequentially clicked the stimulus buttons displayed on the screen and experienced each stimulus at least once for haptic training. In the testing mode, users selected the corresponding button based on the perceived stimulus. Each stimulus was presented twice in a randomized order, with a duration of 1 s per presentation.

The input voltage of the haptic device was fixed at 20 V for the above two experiments. To minimize performance degradation caused by temperature rise during prolonged operation, we allowed 15 s of natural cooling after each directional stimulus. After half of the stimuli, a 1–2 min air cooling period using a fan was applied to the haptic device.

For the user study on perceptual threshold measurement, three stimulus directions were encoded: normal, north‐south shear, and east‐west shear. For each stimulus, five vibration frequencies were tested (1, 25, 50, 75, and 100 Hz), covering all 15 groups. For each group, participants first underwent an ascending sequence. The stimulus intensity (current) started from zero, and participants were asked to report whether they could perceive the stimulus. If the response was No, the PWM duty cycle was increased by 10 (with a maximum value of 255), and the process was repeated until the participant reported Yes. The corresponding PWM duty cycle and current at this point were recorded. Next, a descending sequence was conducted. The PWM duty cycle was increased by 20 from the previously recorded value, and participants were again asked to report whether they could perceive the stimulus. If the response was Yes, the PWM duty cycle was decreased by 5, and the process was repeated until the participant reported No. Finally, the PWM duty cycle at which the participant could no longer perceive the stimulus was increased by 5, and the corresponding current was defined as the perceptual threshold current.

### 4.8 Design of the Wearable Wi‐Fi Remote Control Module

We used the Arduino Uno R4 Wi‐Fi to enable Wi‐Fi communication between the computer and the haptic device. To ensure stable timing of the haptic output, all time‐critical operations (including coil current direction control and PWM signal generation) were executed locally on the Arduino microcontroller. For the power system, an 850 mAh, 11.1 V LiPo battery (Spektrum) was used as the power source. A 10 A rocker switch (Pololu) was used to control the power on and off state of the circuit, and a 3 A panel‐mount circuit breaker (Core Electronics) was integrated to provide short‐circuit protection.

### 4.9 Hand Navigation for Visually Impaired People Using Cutaneous Haptic Cues

We implemented voice input using the speech recognition library and performed object detection on the video stream using the YOLOv11 model. Each target was locked at its center position upon first detection, after which YOLO inference was disabled to reduce computational load. Hand tracking was performed using Media Pipe Hands (Google), with the hand center estimated as the average position of the 21 detected keypoints. For directional haptic cue encoding, the PWM duty cycles of all channels were switched from 0% to 100%, held for 0.5 s, and then immediately reset to 0. For the LEFT and RIGHT cues, synchronous same‐direction encoding was applied, while the UP and DOWN cues were generated using a spatiotemporal sequence with a 0.5 s delay. Vibration cues were generated by alternating the direction of the coil current at 60 Hz for 1.5 s. To avoid excessive heating during prolonged operation of the haptic device, the haptic cues were refreshed every 2 s.

### 4.10 Temperature Measurement of the Electromagnetic Actuator

The XC4494 Analog Temperature Module was used to measure the temperature of the electromagnetic actuator. The module consists of a thermistor and a voltage‐divider resistor. The thermistor was fixed to the side surface of the electromagnetic actuator, and the analog temperature signal was acquired using the Analog Discovery 2. During the experiments, all four coils were driven with identical current magnitudes, while the PWM duty cycle of the input signal was fixed at 100%. The input voltage of the L293D motor driver was set to 5, 10, and 20 V, and periodic current actuation with a cycle period of 1 s was applied to the coils. The ON/OFF time ratio was used to define the proportion of the activation duration within each 1 s operating cycle. For example, a ratio of 20% means that the coils were activated for 200 ms and turned off for the remaining 800 ms.

## Author Contributions

**Jingjing Wan**: conceptualization, investigation, writing – original draft, methodology, validation, visualization, writing – review and editing, software, formal analysis, data curation. **Emanuele Nicotra**: methodology, validation, software, writing – review and editing. **James Davies**: methodology, validation, visualization, writing – review and editing. **Hermione Truong**: validation, writing – review and editing. **Bibhu Sharma**: validation, writing – review and editing. **Tan Huynh**: writing – review and editing. **Quang Anh Nguyen**: validation, writing – review, and editing. **Chi Cong Nguyen**: validation, writing – review and editing. **Sinuo Zhao**: validation, writing – review and editing. **Patrick Pruscino**: validation, writing – review and editing. **Adrienne Ji**: validation, writing – review and editing. **Nigel Hamilton Lovell**: validation, funding acquisition, writing – review and editing, project administration, resources, supervision. **Hoang Phuong Phan**: writing – review and editing, supervision, resources. **Phuoc Thien Phan**: resources, supervision, methodology, validation, visualization, writing – review and editing, project administration. **Kefan Zhu**: validation, visualization, writing – review and editing. **Thanh Nho Do**: conceptualization, investigation, funding acquisition, writing – review and editing, visualization, validation, methodology, formal analysis, project administration, resources, supervision, data curation.

## Ethics Statement

This work was conducted under the approval of the Human Research Ethics Advisory Panel (HREAP) at UNSW, number HC210451.

## Consent

No written consent has been obtained from the patients as there is no patient identifiable data included in this case report/series.

## Conflicts of Interest

The authors declare no conflicts of interest.

## Supporting information




**Supporting File 1**: advs76412‐sup‐0001‐SuppMat.docx.

**Supporting File 2**: advs76412‐sup‐0002‐MovieS1.mp4.

**Supporting File 3**: advs76412‐sup‐0003‐MovieS2.mp4.

**Supporting File 4**: advs76412‐sup‐0004‐MovieS3.mp4.

**Supporting File 5**: advs76412‐sup‐0005‐MovieS4.mp4.

**Supporting File 6**: advs76412‐sup‐0006‐MovieS5.mp4.

**Supporting File 7**: advs76412‐sup‐0007‐MovieS6.mp4.

**Supporting File 8**: advs76412‐sup‐0008‐MovieS7.mp4.

## Data Availability

The data that support the findings of this study are available from the corresponding author upon reasonable request.

## References

[1] J. J. Fleck , Z. A. Zook , J. P. Clark , et al., “Wearable Multi‐sensory Haptic Devices,” Nature Reviews Bioengineering 3 (2025): 288–302, 10.1038/s44222-025-00274-w.

[2] A. Frisoli and D. Leonardis , “Wearable Haptics for Virtual Reality and Beyond,” Nature Reviews Electrical Engineering 1 (2024): 666–679, 10.1038/s44287-024-00089-8.

[3] Y. Huang , K. Yao , J. Li , et al., “Recent Advances in Multi‐mode Haptic Feedback Technologies towards Wearable Interfaces,” Materials Today Physics 22 (2022): 100602, 10.1016/j.mtphys.2021.100602.

[4] Y. Huang , J. Zhou , P. Ke , et al., “Publisher Correction: A Skin‐integrated Multimodal Haptic Interface for Immersive Tactile Feedback,” Nature Electronics 6 (2023): 1041, 10.1038/s41928-023-01115-7.

[5] S. Patel , Z. Rao , M. Yang , and C. Yu , “Wearable Haptic Feedback Interfaces for Augmenting Human Touch,” Advanced Functional Materials 36 (2025): 2417906, 10.1002/adfm.202417906.

[6] P. B. Shull and D. D. Damian , “Haptic Wearables as Sensory Replacement, Sensory Augmentation and Trainer—A Review,” Journal of Neuroengineering and Rehabilitation 12 (2015): 59, 10.1186/s12984-015-0055-z. 26188929 PMC4506766

[7] D. Wang , K. Ohnishi , and W. Xu , “Multimodal Haptic Display for Virtual Reality: A Survey,” IEEE Transactions on Industrial Electronics 67 (2019): 610–623, 10.1109/TIE.2019.2920602.

[8] E. Abdi , D. Kulić , and E. Croft , “Haptics in Teleoperated Medical Interventions: Force Measurement, Haptic Interfaces and Their Influence on User's Performance,” IEEE Transactions on Biomedical Engineering 67 (2020): 3438–3451, 10.1109/TBME.2020.2987603. 32305890

[9] S. C. Lim , H. K. Lee , and J. Park , “Role of Combined Tactile and Kinesthetic Feedback In Minimally Invasive Surgery,” The International Journal of Medical Robotics and Computer Assisted Surgery 11 (2015): 360. 25328100 10.1002/rcs.1625

[10] T. Liu , J. Wang , S. Wong , et al., “A Review on the Form and Complexity of Human–Robot Interaction in the Evolution of Autonomous Surgery,” Advanced Intelligent Systems 6 (2024): 2400197, 10.1002/aisy.202400197.

[11] T. N. Do , T. E. T. Seah , and S. J. Phee , “Design and Control of a Mechatronic Tracheostomy Tube for Automated Tracheal Suctioning,” IEEE Transactions on Biomedical Engineering 63 (2016): 1229–1238, 10.1109/TBME.2015.2491327. 26485352 PMC7186034

[12] I. Bortone , D. Leonardis , N. Mastronicola , et al., “Wearable Haptics and Immersive Virtual Reality Rehabilitation Training in Children with Neuromotor Impairments,” IEEE Transactions on Neural Systems and Rehabilitation Engineering 26 (2018): 1469–1478, 10.1109/TNSRE.2018.2846814. 29985156

[13] S. Handelzalts , G. Ballardini , C. Avraham , M. Pagano , M. Casadio , and I. Nisky , “Integrating Tactile Feedback Technologies into Home‐Based Telerehabilitation: Opportunities and Challenges in Light of COVID‐19 Pandemic,” Frontiers in Neurorobotics 15 (2021): 617636, 10.3389/fnbot.2021.617636. 33679364 PMC7925397

[14] B. Sharma , P. T. Phan , J. Davies , et al., “Soft Upper‐Limb Wearable Robotic Devices: Technology and Applications,” Advanced Intelligent Systems 6 (2024): 2400266, 10.1002/aisy.202400266.

[15] T. T. Hoang , L. Sy , M. Bussu , et al., “A Wearable Soft Fabric Sleeve for Upper Limb Augmentation,” Sensors 21 (2021): 7638, 10.3390/s21227638. 34833719 PMC8620533

[16] T. T. Hoang , C. C. Nguyen , P. T. Phan , et al., “Shape Programmable and Multifunctional Soft Textile Muscles for Wearable and Soft Robotics,” Advanced Intelligent Systems 6 (2024): 2300875, 10.1002/aisy.202300875.

[17] E. Battaglia , J. P. Clark , M. Bianchi , M. G. Catalano , A. Bicchi , and M. K. O'Malley , “Skin Stretch Haptic Feedback to Convey Closure Information in Anthropomorphic, Under‐Actuated Upper Limb Soft Prostheses,” IEEE Transactions on Haptics 12 (2019): 508–520, 10.1109/TOH.2019.2915075. 31071053

[18] Y. H. Jung , J.‐Y. Yoo , A. Vázquez‐Guardado , et al., “A Wireless Haptic Interface for Programmable Patterns of Touch across Large Areas of the Skin,” Nature Electronics 5 (2022): 374–385, 10.1038/s41928-022-00765-3.

[19] F. Barontini , M. G. Catalano , L. Pallottino , B. Leporini , and M. Bianchi , “Integrating Wearable Haptics and Obstacle Avoidance for the Visually Impaired in Indoor Navigation: A User‐Centered Approach,” IEEE Transactions on Haptics 14 (2020): 109–122, 10.1109/TOH.2020.2996748. 32746372

[20] L. Kuang , M. Aggravi , P. R. Giordano , and C. Pacchierotti , “Wearable Cutaneous Device For Applying Position/Location Haptic Feedback In Navigation Applications,” 2022 IEEE Haptics Symposium (HAPTICS) (IEEE, 2022), 1–6.

[21] A. J. Spiers and A. M. Dollar , “Design and Evaluation of Shape‐Changing Haptic Interfaces for Pedestrian Navigation Assistance,” IEEE transactions on haptics 10 (2016): 17–28, 10.1109/TOH.2016.2582481. 27337726

[22] H.‐C. Wang , R. K. Katzschmann , S. Teng , B. Araki , L. Giarré , and D. Rus , “Enabling Independent Navigation for Visually Impaired People Through a Wearable Vision‐Based Feedback System,” 2017 IEEE International Conference On Robotics And Automation (ICRA) , (IEEE, 2017), 6533–6540.

[23] N. Dunkelberger , J. L. Sullivan , J. Bradley , et al., “A Multisensory Approach to Present Phonemes as Language through a Wearable Haptic Device,” IEEE Transactions on Haptics 14 (2020): 188–199, 10.1109/TOH.2020.3009581. 32746381

[24] H. Z. Tan , C. M. Reed , Y. Jiao , et al., “Acquisition of 500 English Words through a TActile Phonemic Sleeve (TAPS),” IEEE Transactions on Haptics 13 (2020): 745–760, 10.1109/TOH.2020.2973135. 32070998

[25] T. T. Hoang , P. T. Phan , M. T. Thai , et al., “Magnetically Engineered Conductivity of Soft Liquid Metal Composites for Robotic, Wearable Electronic, and Medical Applications,” Advanced Intelligent Systems 4 (2022): 2200282, 10.1002/aisy.202200282.

[26] R. S. Dahiya , G. Metta , M. Valle , and G. Sandini , “Tactile Sensing—From Humans to Humanoids,” IEEE transactions on robotics 26 (2009): 1–20, 10.1109/TRO.2009.2033627.

[27] A. Handler and D. D. Ginty , “The Mechanosensory Neurons of Touch and Their Mechanisms of Activation,” Nature Reviews Neuroscience 22 (2021): 521–537, 10.1038/s41583-021-00489-x. 34312536 PMC8485761

[28] K.‐H. Ha , J. Yoo , S. Li , et al., “Full Freedom‐of‐motion Actuators as Advanced Haptic Interfaces,” Science 387 (2025): 1383–1390, 10.1126/science.adt2481. 40146816

[29] F. H. Giraud , S. Joshi , and J. Paik , “Haptigami: A Fingertip Haptic Interface with Vibrotactile and 3‐DoF Cutaneous Force Feedback,” IEEE Transactions on Haptics 15 (2022): 131–141, 10.1109/TOH.2021.3104216. 34379595

[30] D. Leonardis , M. Solazzi , I. Bortone , and A. Frisoli , “A Wearable Fingertip Haptic Device with 3 DoF Asymmetric 3‐RSR Kinematics,” 2015 IEEE World Haptics Conference (WHC) (IEEE, 2015), 388–393.

[31] M. Sarac , T. M. Huh , H. Choi , M. R. Cutkosky , M. Di Luca , and A. M. Okamura , “Perceived Intensities of Normal and Shear Skin Stimuli Using a Wearable Haptic Bracelet,” IEEE Robotics and Automation Letters 7 (2022): 6099–6106, 10.1109/LRA.2021.3140132.

[32] D. Trinitatova and D. Tsetserukou , “Deltatouch: A 3D Haptic Display for Delivering Multimodal Tactile Stimuli at the Palm,” 2019 IEEE World Haptics Conference (WHC) (IEEE, 2019): 73–78.

[33] S. R. Williams , J. M. Suchoski , Z. Chua , and A. M. Okamura , “A 4‐Degree‐of‐Freedom Parallel Origami Haptic Device for Normal, Shear, and Torsion Feedback,” IEEE Robotics and Automation Letters 7 (2022): 3310–3317, 10.1109/LRA.2022.3144798.

[34] A. Raza , W. Hassan , and S. Jeon , “Pneumatically Controlled Wearable Tactile Actuator for Multi‐Modal Haptic Feedback,” IEEE Access 12 (2024): 59485–59499.

[35] Z. Zhakypov and A. M. Okamura , “FingerPrint: A 3‐D Printed Soft Monolithic 4‐Degree‐of‐Freedom Fingertip Haptic Device with Embedded Actuation,” 2022 IEEE 5th International Conference on Soft Robotics (RoboSoft) (IEEE, 2022), 938–944.

[36] K. T. Yoshida , Z. A. Zook , H. Choi , M. Luo , M. K. O'Malley , and A. M. Okamura , “Design and Evaluation of a 3‐DoF Haptic Device for Directional Shear Cues on the Forearm,” IEEE Transactions on Haptics 17 (2024): 483–495, 10.1109/TOH.2024.3365669. 38349838

[37] C. E. Winston , H. Choi , R. Jitosho , et al., “Fourigami: A 4‐Degree‐of‐Freedom, Force‐Controlled, Origami, Finger Pad Haptic Device,” IEEE Transactions on Robotics 41 (2025): 4829–4842, 10.1109/TRO.2025.3593084.

[38] J. Davies , M. T. Thai , T. T. Hoang , et al., “A Stretchable Filament Sensor with Tunable Sensitivity for Wearable Robotics and Healthcare Applications,” Advanced Materials Technologies 8 (2023): 2201453, 10.1002/admt.202201453.

[39] M. T. Thai , P. T. Phan , T. T. Hoang , S. Wong , N. H. Lovell , and T. N. Do , “Advanced Intelligent Systems for Surgical Robotics,” Advanced Intelligent Systems 2 (2020): 1900138, 10.1002/aisy.201900138.

[40] C. C. Nguyen , S. Wong , M. T. Thai , et al., “Advanced User Interfaces for Teleoperated Surgical Robotic Systems,” Advanced Sensor Research 2 (2023): 2200036, 10.1002/adsr.202200036.

[41] M. T. Thai , T. T. Hoang , P. T. Phan , N. H. Lovell , and T. N. Do , “Soft Microtubule Muscle‐Driven 3‐Axis Skin‐Stretch Haptic Devices,” IEEE Access 8 (2020): 157878–157891, 10.1109/ACCESS.2020.3019842.

[42] B. Kang , N. Zavanelli , G. N. Sue , et al., “A Flexible Skin‐Mounted Haptic Interface For Multimodal Cutaneous Feedback,” Nature Electronics 8 (2025): 1.

[43] B. Lim , C. Lee , and D. Hwang , “Development of Embedded Sensor System for 5‐DOF Finger‐Wearable Tactile Interface,” IEEE/ASME Transactions on Mechatronics 26 (2021): 1728–1736, 10.1109/TMECH.2021.3077700.

[44] E. Leroy , R. Hinchet , and H. Shea , “Multimode Hydraulically Amplified Electrostatic Actuators for Wearable Haptics,” Advanced Materials 32 (2020): 2002564, 10.1002/adma.202002564. 32700326

[45] S. Chen , L. Yu , W. Shen , et al., “Multimodal 5‐DOF Stretchable Electromagnetic Actuators toward Haptic Information Delivery,” Advanced Functional Materials 34 (2024): 2314515, 10.1002/adfm.202314515.

[46] H. Kim , H. Yi , H. Lee , and W. Lee ., “HapCube: A Wearable Tactile Device to Provide Tangential and Normal Pseudo‐Force Feedback on a Fingertip,” Proceedings of the 2018 CHI Conference on Human Factors in Computing Systems (IEEE, 2018): 1–13.

[47] Y. Fang , W. Guo , G. Chai , and X. Sheng , “A Lightweight Haptic Interface for Hand‐to‐Object Tasks with Spatiotemporal Displays,” IEEE Transactions on Industrial Electronics 71 (2024): 16255–16263, 10.1109/TIE.2024.3392996.

[48] J. H. Kim , A. Vazquez‐Guardado , H. Luan , et al., “A Wirelessly Programmable, Skin‐integrated Thermo‐Haptic Stimulator System for Virtual Reality,” Proceedings of the National Academy of Sciences 121 (2024): 2404007121, 10.1073/pnas.2404007121. PMC11145186 38768347

[49] K. Bark , J. Wheeler , G. Lee , J. Savall , and M. Cutkosky , “A Wearable Skin Stretch Device for Haptic Feedback,” World Haptics 2009‐Third Joint EuroHaptics conference and Symposium on Haptic Interfaces for Virtual Environment and Teleoperator Systems (IEEE, 2009): 464–469.

[50] A. Ion , E. J. Wang , and P. Baudisch , “Skin Drag Displays,” Proceedings of the 33rd Annual ACM Conference on Human Factors in Computing Systems , (IEEE, 2015), 2501, 10.1145/2702123.2702459.

[51] C. Saudrais , B. Bayle , M. A. Vitrani , and F. Verite , “Skin‐Stretch Haptic Feedback Augmentation Improves Performance in a Simulated Laparoscopic Palpation Task,” IEEE Transactions on Haptics 17 (2024): 578–590, 10.1109/TOH.2024.3363422. 38324441

[52] F. Tokumura , K. Umekage , M. Sado , et al., “Skin Irritation due to Repetitive Application of Adhesive Tape: The Influence of Adhesive Strength and Seasonal Variability,” Skin Research and Technology 11 (2005): 102–106, 10.1111/j.1600-0846.2005.00088.x. 15807807

[53] C. Konya , H. Sanada , J. Sugama , et al., “Skin Injuries Caused by Medical Adhesive Tape in Older People and Associated Factors,” Journal of Clinical Nursing 19 (2010): 1236–1242, 10.1111/j.1365-2702.2009.03168.x. 20345829

[54] J. S. Furyk , C. J. O'Kane , P. J. Aitken , C. J. Banks , and D. A. Kault , “Fast Versus Slow Bandaid Removal: A Randomized Trial,” Medical Journal of Australia 191 (2009): 682–683, 10.5694/j.1326-5377.2009.tb03379.x. 20028307

