# Supplementary material for: A Multimodal Haptic Feedback Interface with Thin‐Film Compliant Mechanism
Source: Adv Sci (Weinh). 2026 Jul 8;13(49):e76412. doi: 10.1002/advs.76412 (PMC13346363; doi:10.1002/advs.76412)
Supplement: Supplementary file 1 [file ADVS-13-e76412-s001.docx]

Supporting Information

**A multimodal haptic feedback interface with thin-film compliant mechanism**

Jingjing Wan ^1^, Emanuele Nicotra ^1^, James Davies ^1^, Kefan Zhu ^1^, Quang Anh Nguyen ^2^, Sinuo Zhao ^2^, Chi Cong Nguyen ^2^, Bibhu Sharma ^1^, Adrienne Ji^1^, Hermione Truong^1^, Patrick Pruscino ^1^, Tan Huynh ^1^, Phuoc Thien Phan ^1^, Hoang Phuong Phan ^2^, Nigel Hamilton Lovell ^1,3^, Thanh Nho Do ^1,3,^*

^1^ School of Biomedical Engineering, Faculty of Engineering, UNSW Sydney, Kensington Campus, Sydney, NSW 2052, Australia

^2^ School of Mechanical and Manufacturing Engineering, Faculty of Engineering, UNSW Sydney, Kensington Campus, Sydney, NSW 2052, Australia

^3^ Tyree Institute of Health Engineering (IHealthE), UNSW Sydney, NSW 2052, Australia

* *Corresponding author*, E-mail: [tn.do@unsw.edu.au](mailto:tn.do@unsw.edu.au)

This PDF file includes:

Figure S1 to S20

Other Supplementary Materials for this manuscript include the following:

Movie S1 to S6

Note S1. theoretical analysis of the static Lorentz force

The electromagnetic force acting on the permanent magnet by a single coil can be calculated using the following equation [1]:

$$\begin{aligned} F_{mag}=V_{r}\left( M\cdot\nabla\right)B\left( x,y,z \right)=V_{r}\left[ \begin{matrix} M_{x}\frac{\delta B_{x}}{\delta x}+M_{y}\frac{\delta B_{x}}{\delta y}+M_{z}\frac{\delta B_{x}}{\delta z} \\ M_{x}\frac{\delta B_{y}}{\delta x}+M_{y}\frac{\delta B_{y}}{\delta y}+M_{z}\frac{\delta B_{y}}{\delta z} \\ M_{x}\frac{\delta B_{z}}{\delta x}+M_{y}\frac{\delta B_{z}}{\delta y}+M_{z}\frac{\delta B_{z}}{\delta z} \end{matrix} \right]\#\left( 1 \right) \end{aligned}$$

Where $F_{mag}$ is the electromagnetic force generated by the coil, $V_{r}$ is the volume of the permanent magnet, $M$ is the magnetization of the magnet, and $B\left( x,y,z \right)$ is the magnetic flux density produced by a single coil. According to the Biot-Savart law, $B\left( x,y,z \right)$ can be calculated using the following equation:

$$\begin{aligned} B\left( x,y,z \right)=\frac{\mu_{0}}{4\pi}\int\frac{Idl\times r^{'}}{\left| r^{'} \right|^{3}}\#\left( 2 \right) \end{aligned}$$

where $I$ is the current in the coil, $dl$ is the directional vector of an infinitesimal coil segment,

$r^{'}$ is the displacement vector, and $\mu_{0}$ is the vacuum permeability.

By combining equations (1) and (2), the magnetic force generated by each of the four coils on the permanent magnet can be computed individually, and the total magnetic force at a fixed magnet position is obtained by summing these four contributions. Figure S3A illustrates the three directional components of the magnetic force produced by Coil 1 on the permanent magnet.

If all coils carry the same current magnitude, we can obtain either a pure shear force or a pure normal force by adjusting the current directions in the four coils. As shown in Figure S3B, when the current directions in all four coils are the same, the resulting electromagnetic force vector sums to a purely normal force:

$$F_{normal}=F_{1z}+F_{2z}+F_{3z}+F_{4z}$$

Where $F_{1z}, F_{2z}, F_{3z}, F_{4z}$ denote the magnitudes of the electromagnetic force components generated by the four coils along the Z-axis.

In contrast, when Coils 1 and 4 have the same current direction, Coils 2 and 3 have the same current direction, and the two coil pairs are driven in opposite directions, the Z-component and X-component of the combined electromagnetic force become zero. In this configuration, the resulting shear force along the Y-axis is given by:

$$F_{shear}=F_{1y}+F_{2y}+F_{3y}+F_{4y}$$

Where $F_{1y}, F_{2y}, F_{3y}, F_{4y}$ denote the magnitudes of the electromagnetic force components generated by the four coils along the Y-axis.

**Note S2. Lumped parameter modelling and dynamic performance analysis**

In Figure S6A, the passive system (TFCM, skin, and air) is represented as a parallel spring-damper system with stiffness $k$ and damping coefficient $c$. The permanent magnet is modelled as a lumped mass $m$, driven by an electromagnetic force $F$.

The dynamic equation of the system is:

$$\begin{aligned} m\ddot{x}+c\dot{x}+kx=F\#\left( 1 \right) \end{aligned}$$

Assuming the external force is the shear electromagnetic force, we performed a parametric sweep of the permanent magnet displacement in COMSOL to obtain the relationship between the electromagnetic force and the magnet displacement under a constant current (Figure S6B). Since the electromagnetic force is proportional to the current, it can be expressed as:

$$\begin{aligned} F_{x}=Ig\left( x \right)=I\left( g_{0}-\alpha x^{2} \right)\#\left( 2 \right) \end{aligned}$$

Assuming the current varies as a sinusoidal signal, we have:

$$\begin{aligned} F=F_{x}=I_{0}\sin\left( \omega t \right)\left( g_{0}-\alpha x^{2} \right)\#\left( 3 \right) \end{aligned}$$

And we can get the final dynamic equation:

$$\begin{aligned} m\ddot{x}+c\dot{x}+kx=I_{0}\sin\left( \omega t \right)\left( g_{0}-\alpha x^{2} \right)\#\left( 4 \right) \end{aligned}$$

Then we performed a parametric sweep of this dynamic equation in MATLAB.

Based on the parametric sweep of the damping coefficient in Figure S6C, we can find that the vibration amplitude decreases with increasing frequency. However, at a given frequency, a higher damping results in a lower amplitude. The greater the system damping, the more energy is dissipated during motion, and therefore the smaller the vibration amplitude. And in Figure S6D, we find that varying only the mass of the permanent magnet does not change the overall trend of the curve; however, at a given frequency, a larger mass results in a smaller vibration amplitude. This is because a larger mass results in greater inertia, requiring more energy from the external force to accelerate the object, thereby leading to a smaller displacement. We hypothesize that the decrease in amplitude with increasing frequency may be influenced by both inertia and damping. Therefore, we separately evaluated the amplitude response under three cases: mass only, damping only, and neither. The result in Figure S6E shows that only when the system has neither inertia nor damping does the amplitude remain independent of frequency, which supports our hypothesis. Interestingly, in the absence of damping, and based on the previously discussed mass sweep results, a system with a larger mass is generally expected to exhibit a lower amplitude at a given frequency. However, as shown in Figure S6E, the curve for $m=8g$ exhibits a higher amplitude than that for $m=0g$ at 20 Hz. This is due to resonance-induced amplification. The system with mass exhibits resonance, with a natural frequency close to 20 Hz, whereas the massless system does not exhibit resonance. This explains why the system with mass shows a higher amplitude at 20 Hz, but its amplitude decreases rapidly and becomes lower than that of the massless system as the frequency increases beyond 20 Hz. In addition, we performed a parametric sweep of stiffness in Figure 6F and found that increasing stiffness leads to a higher resonance frequency but a lower resonance amplitude. Due to the relatively low stiffness of the TFCM in the translational degrees of freedom along the X and Y axes, the corresponding resonance frequencies are below 20 Hz. In contrast, the stiffness in the Z-direction is higher, and therefore a slight resonance behaviour can be observed in the Z-axis vibration characterization. This resonance becomes more pronounced during on-skin measurements, as the skin significantly increases the overall stiffness of the system.

Beyond numerical solutions, the effects of damping and inertia on the amplitude can be analysed using the analytical solution of a simplified model.

If the motion range of the permanent magnet is very small, the dependence of the electromagnetic force on its position can be neglected, leading to a simplified dynamic equation:

$$\begin{aligned} m\ddot{x}+c\dot{x}+kx=g_{0}I_{0}\sin\left( \omega t \right)\#\left( 5 \right) \end{aligned}$$

Then, the steady-state solution of the displacement can be directly obtained:

$$\begin{aligned} X\left( \omega\right)=\frac{g_{0}I_{0}}{\sqrt{\left( k-m\omega^{2} \right)^{2}+\left( c\omega\right)^{2}}}=\frac{g_{0}I_{0}}{\sqrt{m^{2}\omega^{4}+k^{2}-2km\omega^{2}+c^{2}\omega^{2}}}\#\left( 6 \right) \end{aligned}$$

When the damping vanishes and at high frequencies:

$$\begin{aligned} X\left( \omega\right)\approx\frac{g_{0}I_{0}}{m\omega^{2}}\#\left( 7 \right) \end{aligned}$$

In this case, inertia is the key factor causing the amplitude to decrease. And when the inertia vanishes and at high frequencies:

$$\begin{aligned} X\left( \omega\right)\approx\frac{g_{0}I_{0}}{c\omega}\#\left( 8 \right) \end{aligned}$$

Under this condition, damping becomes the dominant factor causing the amplitude decrease.

The analysis above indicates that both inertia and damping may govern the high-frequency amplitude decay. But stiffness appears only in lower-order terms and therefore does not contribute to the amplitude attenuation in the high-frequency regime.

Reducing the mass of the permanent magnet or decreasing the system damping can enhance the amplitude at high frequencies. By keeping the radius of the cylindrical permanent magnet constant, its mass can be reduced by decreasing its height. Although reducing the magnet height lowers its inertia, it also leads to a reduction in the electromagnetic force. Therefore, we performed simulations in COMSOL to evaluate the effect of magnet height on the electromagnetic force acting on the permanent magnet. As shown in Figure S7, we find that the electromagnetic force decreases more slowly than the inertia as the mass is reduced. For example, when the magnet height (2 mm) is reduced to 40% of its initial value (5 mm), the inertia is correspondingly reduced to 40% of its original value, whereas the electromagnetic force remains at 57% in the shear direction and 52% in the normal direction. Based on equation (7), the high-frequency amplitude is therefore estimated to increase by a factor of 1.425 in the shear direction and 1.3 in the normal direction. These results provide preliminary evidence that reducing the magnet height can improve the high-frequency amplitude of the device.

In addition, the high-frequency response can be further enhanced by reducing the system damping, for example, by minimizing the contact area and preload between the tactor and the skin, and by employing low-loss materials such as polyimide (PI) films for fabricating the TFCM.

The lumped-parameter model can also be used to analyse another phenomenon: underdamped oscillation after the electromagnetic force is reduced to zero. During quasi-static shear motion of the haptic device, when the driving current is turned off, the electromagnetic force disappears abruptly, while the permanent magnet still has non-zero velocity, acceleration, and displacement. The magnet is then driven back toward the equilibrium position by the restoring force of the compliant mechanism. However, due to inertia, it does not stop exactly at the equilibrium position but instead overshoots and exhibits a decaying oscillation.

Once the driving current is turned off, the electromagnetic force vanishes abruptly, and we can get:

$$\begin{aligned} m\ddot{x}+c\dot{x}+kx=0\#\left( 9 \right) \end{aligned}$$

Then we define natural angular frequency:

$$\begin{aligned} \omega_{n}=\sqrt{\frac{k}{m}}\#\left( 10 \right) \end{aligned}$$

And damping ratio:

$$\begin{aligned} \zeta=\frac{c}{2\sqrt{km}}\#\left( 11 \right) \end{aligned}$$

Equation (9) then becomes

$$\begin{aligned} \ddot{x}+2\zeta\omega_{n}\dot{x}+{\omega_{n}}^{2}x=0\#\left( 12 \right) \end{aligned}$$

When $0<\zeta<1$, the system is underdamped. And the damped frequency is:

$$\omega_{d}=\omega_{n}\sqrt{1-\zeta^{2}}$$

Let the permanent magnet have a displacement $x_{0}$ and a velocity $v_{0}$ at the instant when the electromagnetic force vanishes. We can then get the underdamped oscillation equation:

$$\begin{aligned} x\left( t \right)=e^{-\zeta\omega_{n}t}\left[ x_{0}\cos\omega_{d}t+\frac{v_{0}+\zeta\omega_{n}x_{0}}{\omega_{d}}\sin\omega_{d}t \right]\#\left( 13 \right) \end{aligned}$$

And the amplitude of the underdamped oscillation is:

$$\begin{aligned} A=\sqrt{{x_{0}}^{2}+\left( \frac{v_{0}+\zeta\omega_{n}x_{0}}{\omega_{d}} \right)^{2}}\#\left( 14 \right) \end{aligned}$$

According to equation (13) and (14), the oscillation amplitude of the permanent magnet after the current is turned off is positively correlated with $x_{0}$ and $v_{0}$. Increasing the damping accelerates the decay of the oscillation, and with sufficiently large damping ($\zeta>1$), the system becomes overdamped, thereby eliminating noticeable oscillatory behaviour. This explains why the device exhibits the underdamped oscillation in free motion, whereas no clear or regular oscillatory behaviour is observed during on-skin operation (Figure S8). Therefore, the shear haptic cues are not expected to be significantly affected by such underdamped oscillations when applied to the skin.

To suppress the underdamped oscillation under the free-motion condition, the mass of the permanent magnet can be reduced, or the ramp-down duration of the PWM duty cycle can be extended to ensure sufficient deceleration time. In addition, a brief and low-amplitude reverse pulse can be applied immediately before the duty cycle reaches zero to compensate for the residual velocity $v_{0}$.

Figure S1. Effects of PET film thickness (0.075 mm, 0.125 mm, and 0.2 mm), film material (polyethylene terephthalate (PET), polyimide (PI), and polyethylene naphthalate (PEN)), and PET flexible beam width (2.5 mm, 5 mm, and 7.5 mm) on the translational and torsional stiffness of the TFCM along the X-axis. In the thickness study, the flexible beam width was fixed at 5 mm and all samples were fabricated using PET films. In the width study, the flexible beam thickness was fixed at 0.125 mm and all samples were fabricated using PET films. In the material study, the flexible beam width and thickness were fixed at 5 mm and 0.125 mm, respectively.

Figure S2. Reaction force measurements for cyclic loading tests of the five degrees of freedom. The translational displacement and rotational angle were set to 4 mm and 5°, respectively, with 10,000 loading cycles applied at a frequency of 2 Hz.

Figure S3. Theoretical analysis of the electromagnetic force on the permanent magnet. (A) Electromagnetic force on the permanent magnet generated by a single coil. (B) Pure shear and normal forces obtained from the vector summation of four independent electromagnetic forces.

Figure S4. Force-to-power efficiency of the electromagnetic actuator in the shear and normal modes versus current.

Figure S5. The temperature change of the haptic device under different input coil currents and ON/OFF time ratios.

Figure S6. Lumped parameter modelling and dynamic performance analysis of the haptic module. (A) Lumped parameter mass-spring-damper modal of our device. (B) Relationship between the shear EM force and the position of the permanent magnet. (C) Parametric sweep of the damping coefficient from 0 to 1. (D) Parametric sweep of the permanent magnet mass from 8 g to 58 g. (E) Dynamic performance of the haptic module under zero-mass or zero-damping conditions. (F) Parametric sweep of the stiffness coefficient from 0 to 1210 N/m.

Figure S7. Effect of the permanent magnet height (mass) on the shear and normal electromagnetic forces.

Figure S8. Comparison of the displacement responses of the haptic device after the electromagnetic force is turned off under free-motion and on-skin conditions.

Figure S9. Quasi-static displacement output and dynamic response of the haptic module under different loads.

Figure S10. Characterization of the Z-direction displacement of the haptic device on the skin. (A) Schematic of the experimental setup for characterizing the Z-direction displacement using a laser displacement sensor. (B) Quasi-static displacement in the normal direction. (C) Peak-to-peak vibration amplitude in the Z direction under different input frequencies.

Figure S11. Displacement recording of the haptic device during 40 min of high-amplitude shear vibration (5 Hz).

Figure S12. Measurement of the parasitic torsional angle during shear motion using the Tracker software. A reference vector A was marked, and its angular change was recorded.

Figure S13. Threshold currents under different body locations (finger and forearm), stimulation modes (normal, north-south shear, east-west shear), and vibration frequencies (static and 25-100 Hz).

Figure S14. Wireless Wi-Fi control system and power supply for the haptic device.

Figure S15. Details of the TFCM fabrication. (A) Cut and fold the flexible beams. (B) Dimensions of the two flexible beams.

Figure S16. The setup for characterising the force-displacement behaviour of TFCM.

Figure S17. The relationship between the input duty cycle and the four output currents under the different voltages.

Figure S18. The setup for measuring the blocked force of the electromagnetic actuator.

Figure S19. Displacement measurement of the haptic device using the laser displacement sensor.

Figure S20. Experimental setup and graphical user interface (GUI) for the user study.

Figure S21. Peeling force degradation of adhesive tapes after two attachment-removal cycles. The maximum 90° peeling forces of Tesa Tape and Walker Tape (medical) were measured on the dorsal hand skin during the first and second attachment-removal cycles. Both tapes exhibited reduced peeling force after the first removal, indicating degraded re-adhesion capability.

**References**

[1]	B. V. Johnson, S. Chowdhury, D. J. Cappelleri,"Local magnetic field design and characterization for independent closed-loop control of multiple mobile microrobots," *IEEE/ASME Transactions on Mechatronics 25*, no. 2 (2020): 526-534. https://doi.org/10.1109/TMECH.2020.2969074
